# Zero‐Valent Ruthenium‐Anchored Nanozymes Remodel Tumor Microenvironment for Malignant Tumor Treatment in Combination With αPD‐L1 Immunotherapy

**DOI:** 10.1002/exp2.70209

**Published:** 2026-08-10

**Authors:** Xihang Ren, Zhaoxu Meng, Liyuan Guan, Yanzhu Wang, Zhenjun Chen, Zhou Li, He Lian, Yiping Mu

**Affiliations:** ^1^ Wuya College of Innovation Shenyang Pharmaceutical University Shenyang China; ^2^ Department of Biomedical Engineering School of Medical Devices Shenyang Pharmaceutical University Shenyang China; ^3^ Central Hospital Affiliated to Shenyang Medical College Shenyang China; ^4^ Vita Tech Innovation Center Tsinghua Changgung Hospital School of Clinical Medicine Tsinghua Medicine Tsinghua University Beijing China; ^5^ School of Biomedical Engineering Tsinghua Medicine Tsinghua University Beijing China; ^6^ Beijing Key Laboratory of Clinical Innovation and Translation for Active Implantable and Interventional Medical Devices Tsinghua Changgung Hospital Beijing China

**Keywords:** immunotherapy, nanozymes, oxidative stress, three‐enzyme catalysis, tumor microenvironment

## Abstract

The complex tumor microenvironment (TME) includes high concentrations of hydrogen peroxide, high expression of reduced glutathione (GSH), hypoxia, and the resulting immunosuppression from the combination of these features, posing major challenges in tumor therapy. Although nanozymes have demonstrated remarkable potential in treating malignant tumors, nanozymes with zero‐valent metal doping that can simultaneously catalyze the above three indicators to reverse the TME are still limited. Herein, zero‐valent ruthenium was anchored on iron‐based materials to obtain FeRu nanozymes with ruthenium as the “electron bridge” and iron as the “REDOX center” to enhance the electron transfer efficiency. Its hollow structure significantly exposes more catalytic active sites. Density functional theory calculations theoretically explain the potential triple catalytic and electron transfer mechanisms of the nanozyme. The catalase‐like, peroxidase‐like, and GSHOx‐like activities of FeRu nanozymes rapidly generate a large amount of oxygen and hydroxyl radicals, significantly consume GSH, and disrupt the redox balance in tumor cells. These in situ catalytic reactions lead to cell damage, necrosis in malignant tumors, release of damage‐associated molecular patterns, and activation of the adaptive immune response. This work also performs a combined application of anti‐programmed cell death‐ligand 1 and FeRu nanozymes to further enhance the efficacy of immunotherapy, thus providing a new perspective for future cancer therapy.

## Graphical abstract

The tumor microenvironment featuring abundant hydrogen peroxide, high glutathione, and hypoxia brings huge obstacles to tumor treatment. In this study, zero‐valent metal‐doped nanozymes (FeRu) can simultaneously regulate these three factors due to the optimized electron transfer and hollow structure: breaking the redox balance of tumor cells, triggering tumor cell death, and activating anti‐tumor immunity. The combined treatment with anti‐programmed cell death‐ligand 1 further enhanced the efficacy of immunotherapy, providing a new strategy for cancer treatment.

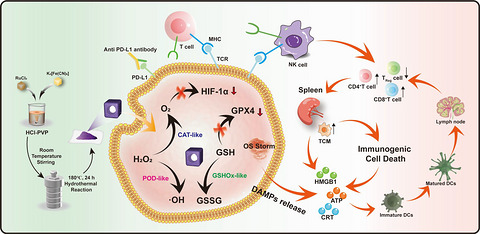

## 1 Introduction

The tumor microenvironment (TME) is a highly complex and precisely coordinated “pathological ecosystem” dominated by tumor cells as the core. This environment also includes tumor‐associated stromal cells, immune cells, etc. They build a perilous survival system through the “malignant signal molecules” they secrete, such as cytokines and chemokines, as well as the “structural network” of extracellular matrix [1]. The main characteristics of TME are a mildly acidic environment, hypoxia, excess hydrogen peroxide (H_2_O_2_), high expression of glutathione (GSH), and immunosuppression.

H_2_O_2_ is a type of reactive oxygen species (ROS) in tumor cells and also an intracellular messenger molecule [2]. H_2_O_2_ and its metabolites can affect the redox state of cells, thereby influencing the metabolic processes of tumor cells [3]. Due to the rapid proliferation of tumor cells, the concentration of H_2_O_2_ is often higher than that of normal cells [4]. Tumor cells can adaptively upregulate concentrations of GSH to reduce the adverse effects caused by high concentrations of H_2_O_2_ [5, 6]. Based on this, therapeutic methods that utilize or target high H_2_O_2_ concentrations in tumors are emerging [7]. However, the oxidation capacity of H_2_O_2_ is relatively weak, and it is difficult to cause an oxidative stress storm in tumors [8]. Therefore, how to convert abundant H_2_O_2_ in tumors into highly toxic ROS (such as ·OH) or O_2_ is a key to treating tumors.

Most solid tumors are found to be hypoxic, with pO_2_ pressure <8 mmHg [9]. The imbalance between low oxygen supply and high oxygen consumption by tumor cells leads to a hypoxic tumor environment [10], triggering overexpression of hypoxia‐inducible factor‐1α (HIF‐1α). It enables tumor cells to maintain rapid proliferation and metabolism [11] and helps tumor cells evade the toxic effects of tumor‐infiltrating T‐lymphocyte‐mediated killing [12]. A previous study has shown that blocking the transcriptional activity of HIF‐1α can significantly inhibit the growth of B16 melanoma [13], and increasing oxygen supply has also been demonstrated to suppress the expression of HIF‐1α [14].

In recent years, the application of nanozymes to reverse the TME has become an important aspect of tumor treatment research [15]. Nanozymes are a type of nanomaterial with enzyme‐like catalytic properties [16]. They can effectively combine the characteristics of traditional chemical catalysts and biocatalysts [17, 18]. Notably, nanozymes can facilitate the in situ production of therapeutic substances for tumor elimination [19], such as ROS‐mediated therapy [20]. Heteronuclear bimetallic nanozymes with rational electronic modulation have been recognized as an effective strategy to boost multi‐catalytic performance in tumor therapy, which provides theoretical guidance for designing zero‐valent Ru‐anchored iron‐based nanozymes [21].

Ruthenium (Ru) is a rare precious metal, and its electronic structure is similar to that of iron. Its catalytic activity in various processes such as oxygen evolution and hydrogen evolution is much higher than that of iron [22, 23]. Therefore, Ru‐based materials are regarded as promising candidates for nanozymes [24]. Ru nanoparticles (NPs) could be anchored on various supports, such as carbon, carbon composites, metals, and semi‐metal‐based materials, and exhibit excellent catalytic properties [25, 26]. For instance, Liu et al. encapsulated Ru NPs into a carbon shell electrocatalyst [27]. The large specific surface area of the hollow carbon sphere carrier (288 m^2^g^−1^) not only prevents the accumulation of metal Ru but also demonstrates remarkable structural and catalytic stability. In addition, the introduction of heteroatoms (N, P, S, and B) into the carbon carrier was found to improve the catalytic performance [28]. Besides, precursors containing C and N sources can be utilized to enhance the dispersibility of Ru metal [29], which also provides a reference for the construction of nanozymes in this study. Furthermore, Lu et al. successfully constructed a multifunctional nanoplatform integrating photothermal, photodynamic, chemodynamic, and immunotherapy through ruthenium‐doped carbon dots, and for the first time discovered its ability to induce PANoptosis and effectively suppress tumor metastasis, providing a promising new strategy for efficient cancer therapy [30]. Another study successfully stabilized ruthenium single‐atom centers in an LDH‐MXene hybrid carrier, achieving synergistic photothermal and catalytic effects and addressing the stability and efficiency challenges of single‐atom catalysts in biological applications [31]. These ruthenium‐based platforms demonstrate remarkable potential in advancing both oncotherapy and catalytic applications.

Another major feature of TME is the immunosuppressive microenvironment; it not only promotes tumor progression but also severely limits the efficacy of emerging immunotherapies, particularly immune checkpoint inhibitors (ICIs) such as anti‐programmed death‐ligand 1 (αPD‐L1) antibodies [32]. Although ICIs can reactivate T cells to attack tumors, their success is often contingent upon a pre‐existing tumor‐infiltrating lymphocyte population and a favorable, immunological “hot” TME. However, in many “cold” tumors with poor immunogenicity, the lack of T cell infiltration and the dominance of immunosuppressive factors render ICIs ineffective [33]. Therefore, strategies that can trigger robust tumor cell death and simultaneously stimulate innate and adaptive immune responses – a process known as “cold” tumors turning “hot” – are highly sought after to enhance ICI therapy [34]. Related studies have demonstrated that novel nanoscale drug delivery systems can improve tumor enrichment and reduce systemic toxicity of ICIs. Various nanocarriers and biodegradable scaffold delivery platforms are expected to break through the limitations of current mUC immunotherapy [35]. In this case, in situ catalytic nanomedicine remodeling of the TME offers a promising avenue to enhance the reactivity of tumors to ICIs.

**SCHEME 1:**
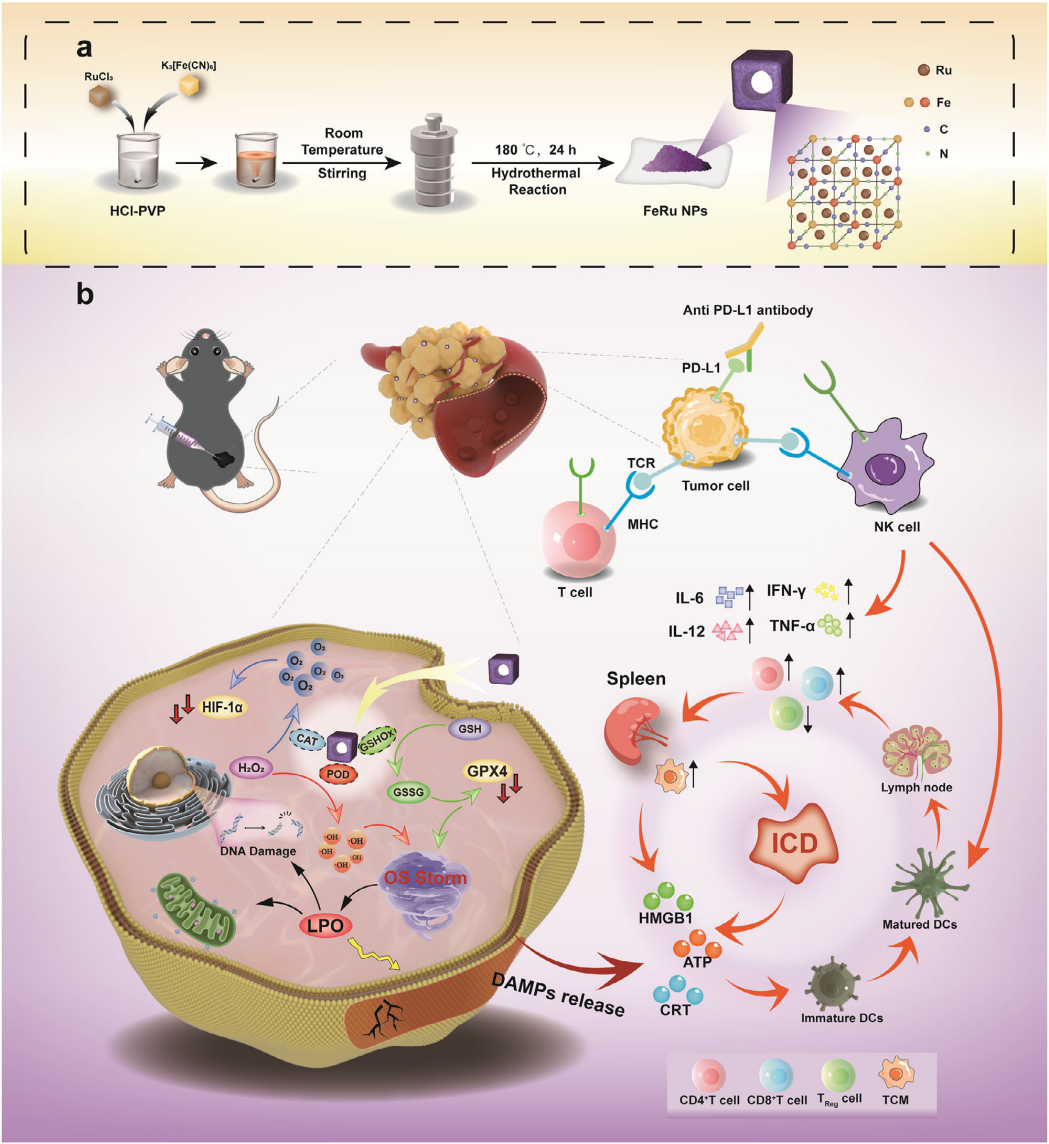
Schematic illustration of (a) the synthetic processes of FeRu nanozymes and (b) the mechanism of three‐enzyme activity catalysis and immune‐activated therapy in the tumor microenvironment.

Metal–organic frameworks (MOFs, as a major category of metal‐based nanocarriers) can controllably release intracellular metal ions to trigger metalloptosis via Fenton/Fenton‐like catalytic reactions. Multifunctional MOF nanozymes integrating metalloptosis, phototherapy, and immunotherapy represent the mainstream direction of next‐generation tumor therapeutic nanomaterials [36]. Herein, the one‐pot method was used to synthesize zero‐valent Ru‐anchored hollow iron‐based nanozyme (FeRu), using ruthenium as the “electron bridge” and iron as the “REDOX center” to enhance electron transfer and catalytic efficiency. The hollow structure of FeRu nanozymes increases its specific surface area, exposing more active sites and thus enhancing its catalytic activity [22]. FeRu nanozymes could simultaneously possess triple enzyme‐like activities under TME conditions: CAT‐like, peroxidase (POD)‐like, and GSHOx‐like activities. First, the catalase‐like activity efficiently catalyzes H_2_O_2_ to generate a large amount of oxygen, reverses the hypoxic state at the tumor site, and downregulates the expression of HIF‐1α in B16 cells. Second, the POD‐like activity of FeRu nanozymes effectively converts H_2_O_2_ to highly toxic ·OH, enhancing oxidative stress in tumor cells [37]. Third, GSHOx‐like activity accelerates the oxidation of GSH and decreases intracellular GSH levels, leading to the deactivation of glutathione peroxidase 4 (GPX4). The three enzyme‐like catalytic activities play distinct roles in the TME and complement each other, triggering oxidative stress storms, significantly reducing mitochondrial membrane potential, and causing DNA damage and cell death. The damage‐associated molecular patterns (DAMPs) are then released to upregulate the infiltration of various immune cells (mainly CD8^+^ T cells), activate adaptive immune responses, and reshape the tumor immunosuppressive microenvironment (Scheme 1).

Programmed cell death receptor 1 (PD‐1) is an inhibitory immune checkpoint. It regulates the immune response and maintains the balance of the immune system by binding to programmed cell death ligand 1 (PD‐L1). However, when PD‐L1 on the surface of tumor cells binds to PD‐1 on the surface of immune cells, it inhibits the activity and function of the latter, forming an immunosuppressive TME and leading to immune escape of tumor cells and tumor progression [38]. The ICI αPD‐L1 can block the binding between PD‐L1 on the surface of tumor cells and PD‐1 on the surface of T cells, enhancing the ability of T cells to kill tumor cells [39]. However, the therapeutic effect of a single inhibitor is limited, mainly due to the presence of other ways for tumors to escape and other immunosuppressive factors [40]. Herein, we combined αPD‐L1 and FeRu nanozymes, attempting to maximize the activation of immunity at the tumor site through combination therapy.

In summary, this study proposes a zero‐valent Ru‐anchored hollow Fe‐based nanozyme, which utilizes the electron transfer effect of zero‐valent metals to enhance in situ catalytic therapy. By combining with αPD‐L1, a new solution strategy is provided for reversing the tumor immunosuppressive microenvironment and treating malignant tumors.

## 2 Results and Discussion

### 2.1 Synthesis and Characterization of FeRu Nanozymes

**FIGURE 1:**
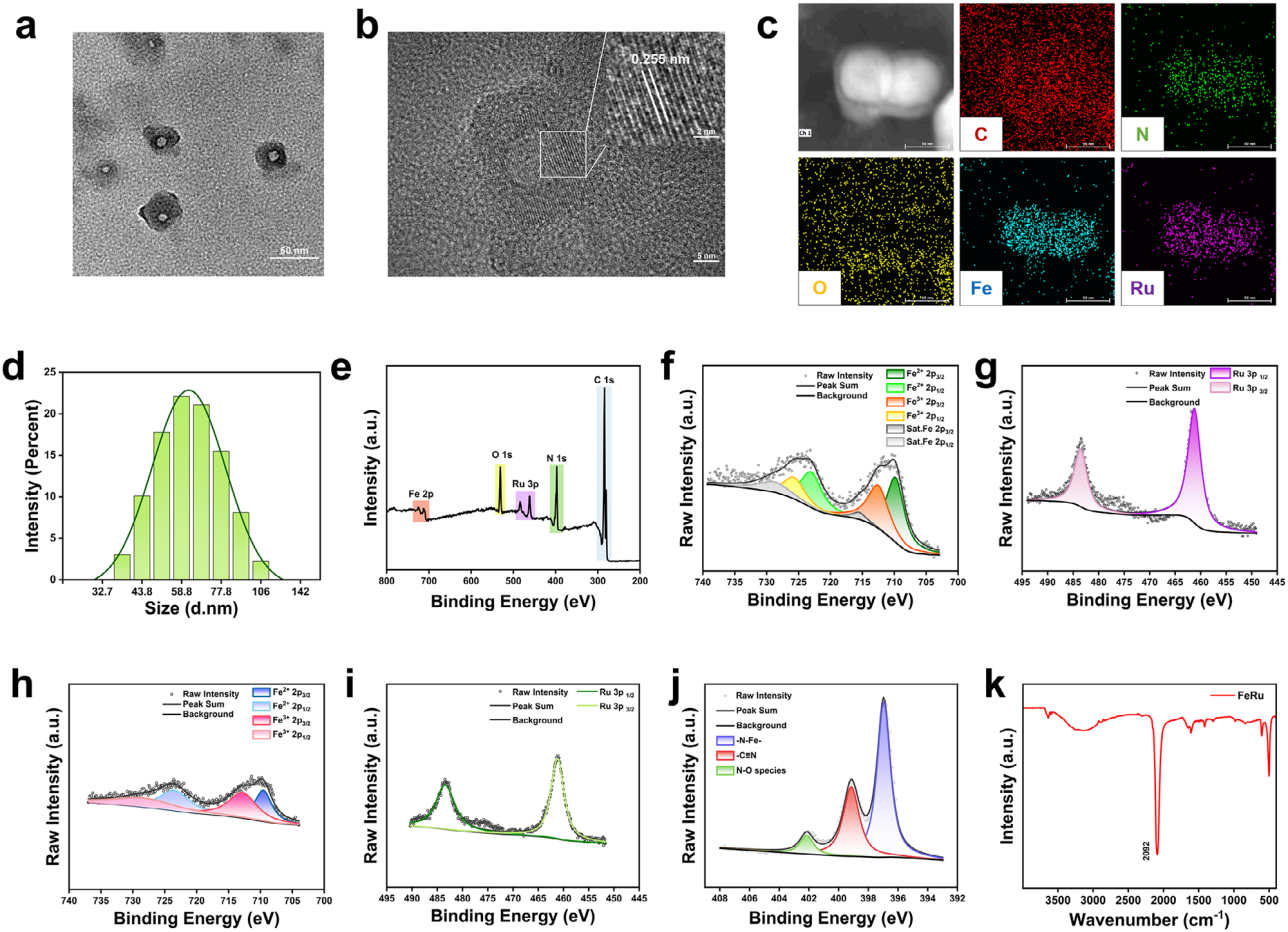
Structural and compositional characterizations of FeRu nanozymes. (a) TEM images of FeRu nanozymes. (b) The HRTEM of FeRu nanozymes. (c) The elemental mapping of C, N, O, Fe, and Ru of FeRu nanozymes. (d) The average size of FeRu nanozymes by DLS. (e) Survey XPS spectra of FeRu nanozymes. The XPS spectra of (f) Fe 2p, (g) Ru 3p, and (j) N 1s in FeRu nanozymes before the catalytic reaction. The XPS spectra of (h) Fe 2p and (i) Ru 3p in FeRu nanozymes after the catalytic reaction. (k) FT‐IR spectra of FeRu nanozymes.

The preparation process is shown in Scheme 1a. Hollow iron Ru nanozymes with special structures were obtained by referring to the classical one‐pot method [41]. As shown in Figure 1a, the transmission electron microscopy (TEM) image revealed that FeRu nanozymes had a three‐dimensional hollow cubo‐like frame. The formation of the distinctive hollow structure can be attributed to the Ostwald ripening process mediated by coordinating polymer during the hydrothermal treatment process [42, 43]. Briefly, the rapid coordination between Ru^3+^ and [Fe(CN)_6_]^4−^ first generates solid coordination polymer cubes in the presence of PVP. At higher temperature and pressure, the ripening process was activated. The higher energy interior of the cubes dissolved preferentially, then the dissolved substances subsequently recrystallized on the more stable outer surface, leading to the evolution from solid to hollow structure [44]. This mechanism has been well‐established for the synthesis of hollow micro‐nanostructures derived from metal‐organic frameworks or coordination polymers. Figure 1b shows a high‐resolution TEM (HRTEM) image taken at 1500 K magnification and 200 kV high voltage. The measured crystal surface spacing was *d* = 0.255 nm. The elemental mapping in Figure 1c demonstrates the uniform distribution of Fe, Ru, C, and N elements in the cubic structure. The full‐spectrum analysis of the elemental composition of FeRu nanozymes is shown in Figure S1, indicating the formation of FeRu nanozymes and that the ratio of Fe and Ru elements in the nanozymes is about 2:1. We then measured the size of the nanozymes by the DLS method. The result shows that the hydrodynamic particle size of the nanozymes was 63.82 nm (Figure 1d) and remained stable for a week (Figure S2). In addition, the zeta potential of nanozymes was −33.4 mV (Figure S3). The structure of the sample was further analyzed by X‐ray diffraction. The sample exhibited a face‐centered cubic structure with five main peaks near 17.0°, 24.2°, 34.4°, 38.5°, and 42.5°, corresponding to (200), (220), (400), (420), and (422) crystal planes, respectively (Figure S4). A comparison of the crystal face spacing showed that 400 crystal faces were exposed in the HRTEM image. Furthermore, the XPS spectra confirmed the presence of Fe, Ru, C, N, O, and Cl elements in the FeRu nanozymes (Figure 1e). Due to the presence of HCl, water, and PVP in the preparation process, the XPS spectra of Cl 1s and O 1s were not considered for analysis. In the Fe 2p spectrum, two pairs of peaks at 723.18/709.88 and 725.98/712.68 eV were assigned to Fe^2+^ and Fe^3+^, respectively (Figure 1f). By integrating the peak areas of the two valence states, the ratio of Fe^2+^/Fe^3+^ in the sample was found to be 1.08. Notably, the 3d peak of Ru overlapped with the C1s peak in the XPS spectrum. The peaks at 461.4 and 483.6 eV in the Ru 3p diagram shifted slightly toward lower binding energy compared with metallic Ru reported in the literature [45] (Figure 1g). Since the change in binding energy is determined by electron transfer in the outer layer, this negative shift in binding energy indicates that a portion of electrons may be flowing from Fe^2+/3+^ to Ru. This also confirmed that Ru in the nanozymes was mainly in the zero‐valent metallic state (Ru 0). After the catalytic reaction, the Fe^2+^/Fe^3+^ ratio in the FeRu nanozymes decreased from 1.08 (as‐synthesized) to 0.73 (post‐catalysis) (Figure 1h). This indicates that a portion of Fe^2+^ was oxidized to Fe^3+^ during the catalytic process, directly proving that iron acts as the primary redox‐active center and participates directly in electron transfer. The consumption of Fe^2+^ and accumulation of Fe^3+^ confirm its key role as an electron donor in the catalytic cycle. In contrast, as shown in Figure 1i, Ru remained in the zero‐valent metallic state before and after catalysis, with no detectable Ru oxide peaks. The stability of Ru^0^ suggests that ruthenium does not undergo a traditional redox cycle like iron. Therefore, we propose that metallic ruthenium anchored on the iron‐based framework functions as an efficient “electron channel” or “electron reservoir.” Due to its excellent conductivity and high electron density, Ru° facilitates rapid electron transfer from Fe^2+^ to adsorbed reactants (e.g., H_2_O_2_, GSH), thereby accelerating reaction kinetics. Moreover, the presence of Ru^0^ may promote the adsorption and activation of reactant molecules on its surface and work in synergy with the iron centers to lower the overall reaction energy barrier. In summary, the post‐catalytic XPS data clearly illustrate a synergistic mechanism: iron serves as a “sacrificial redox center,” driving the reaction through valence state changes, while Ru^0^ acts as a stable “electron bridge,” enhancing overall electron transfer efficiency and stabilizing the catalytic cycle. The different roles and the synergistic interaction between the two metals are the keys to the high and sustained triple‐enzyme activity of FeRu nanozymes.

According to the fitting analysis of the XPS pattern of the N 1s peak, 399.16 and 396.90 eV in N 1s were attributed to −C≡N and ‐N‐Fe‐, respectively (Figure 1j). Furthermore, the weak peak observed at 402.15 eV can be attributed to either nitrogen oxides (such as nitroso or nitro groups) or surface‐adsorbed nitrogen‐containing molecules like NO_2_ or NH_3_. Fourier transform infrared analysis was also conducted. As shown in Figure 1k, a strong absorption peak at 2088.8 cm^−1^ was clearly observed, revealing the vibration of the −C≡N chemical bond.

### 2.2 In Vitro Evaluations of the Multi‐Enzymatic Catalytic Activities of FeRu Nanozymes

**FIGURE 2:**
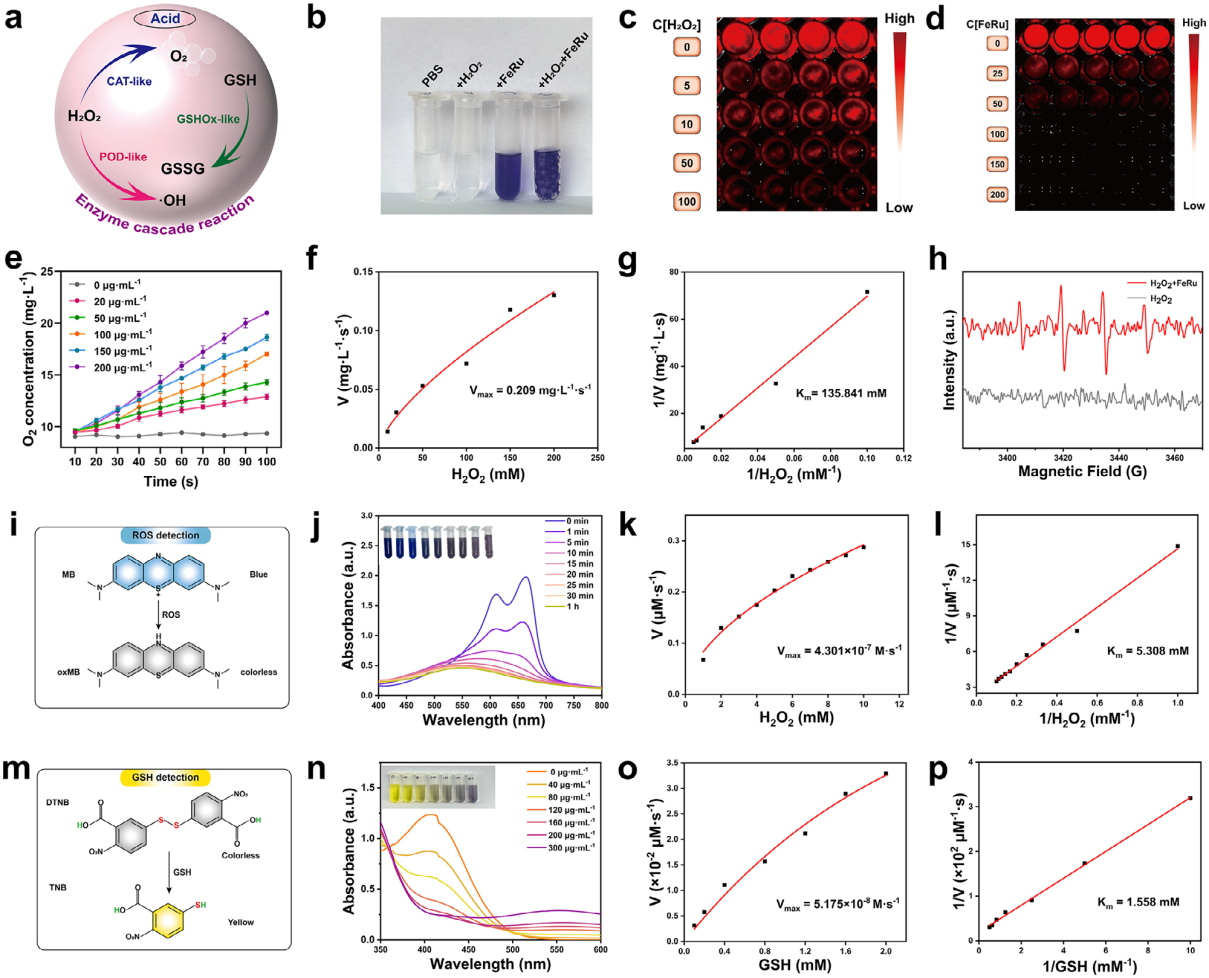
Multienzymatic catalytic activity evaluations of FeRu nanozymes. (a) The three catalytic activities of FeRu nanozymes. (b) Digital photos of bubble formation on tube walls in different groups of solutions. (c) Detection of FeRu nanozymes‐catalyzed O_2_ generation with different concentrations of H_2_O_2_. (d) Detection of O_2_ generation with different concentrations of FeRu nanozymes. (e) The O_2_ concentrations catalyzed by FeRu nanozymes of different concentrations. (f) Michaelis–Menten curves and (g) Lineweaver–Burk plots of CAT‐like activity. (h) ESR spectroscopy to confirm the generation of ·OH by utilizing DMPO as the trapping agent. (i) The reaction principle of MB as the ·OH probe. (j) ·OH production ability of FeRu nanozymes at different times. (k) Michaelis–Menten curves and (l) Lineweaver–Burk plots of POD‐like activity. (m) Reaction principle of DTNB as the GSH probe. (n) GSH‐consuming ability of FeRu nanozymes at different concentrations. (o) Michaelis–Menten curves and (p) Lineweaver–Burk plots of GSHOx‐like activity (*p < 0.05, **p < 0.01, ***p < 0.001, ****p < 0.0001, ns, no significant).

Figure 2a shows the three catalytic activities of FeRu nanozymes. We conducted a comprehensive study of their three‐enzyme catalytic activity. First, the consumption of H_2_O_2_ in the system was tested in a weakly acidic condition (phosphate‐buffered saline (PBS), pH 6.5). The absorbance of ammonium titanium oxalate (A_365 nm_) gradually decreased with increases in FeRu nanozyme concentrations. In addition, the absorbance at 365 nm gradually decreased over time, indicating the significant consumption of H_2_O_2_ in the solution (Figure S5). Similar to natural catalase, FeRu nanozymes could effectively catalyze H_2_O_2_ to generate O_2_. As shown in Figure 2b, in a slightly acidic PBS solution (pH 6.5), H_2_O_2_ and FeRu nanozymes alone did not produce O_2_ bubbles. However, under the catalytic conditions of both H_2_O_2_ and FeRu nanozymes, a large number of bubbles gradually appeared on the tube wall within 10 min. This phenomenon indicates that FeRu nanozymes can effectively exhibit CAT‐like activity and catalyze the decomposition of H_2_O_2_ to produce O_2_. We then detected the production of O_2_ in a solution using [Ru(pdd)_3_]Cl_2_ as a fluorescent probe. More O_2_ was produced with increases in H_2_O_2_ concentrations, leading to weaker red [Ru(pdd)_3_]Cl_2_ fluorescence (Figure 2c), and Figure S6a shows the relative fluorescence value. Similarly, higher concentrations of FeRu nanozymes resulted in more O_2_ production, significantly quenching the red [Ru(pdd)_3_]Cl_2_ fluorescence (Figure 2d and Figure S6b). This phenomenon indicates that the FeRu nanozymes exhibited outstanding CAT‐like activity in weakly acidic conditions. Figure 2e is the curve of O_2_ concentration produced by different concentrations of FeRu nanozymes catalyzed by a dissolved oxygen meter. With the increase of the concentration of nanozymes, the dissolved O_2_ produced in the solution increased significantly at the same time. The curve without nanozymes (gray curve) remains stable, indicating that no excess O_2_ is produced. Furthermore, the nanozyme maintained excellent CAT‐like activity in a biomimetic medium containing 10% FBS, and the oxygen generation increased in a concentration‐dependent manner (Figure S7), which strongly supported the subsequent in vivo studies. Subsequently, we evaluated the enzymatic kinetics of FeRu nanozymes. The CAT‐like enzymatic kinetics of FeRu nanozymes were evaluated by the concentration of O_2_ in the dissolved oxygen meter. The *K_M_* value and the maximum reaction rate (*V*_max_) were 135.841 mM and 0.209 mg·L^−1^·s^−1^, respectively (Figure 2f,g). Table S1 summarizes the CAT‐like catalysis kinetics parameters of natural enzymes and some similar nanozymes. It can be seen that although FeRu nanozymes have a relatively poor affinity for substrates, they have a better catalytic rate than other enzymes.

Then, we further assessed the POD‐like activity of the FeRu nanozymes. The hydroxyl radicals (·OH) generated in enzyme‐catalyzed reactions was also examined using electron spin resonance (ESR) spectra. No radicals were observed in pure H_2_O_2_ solution (Figure 2h). However, the distinct 1:2:2:1 characteristic quadruple signal from the H_2_O_2_ + FeRu group showed the production of ·OH, which is thought to be from the rapid transition of H_2_O_2_ to ·OH due to the POD‐like activity of FeRu nanozymes. Then we detected ·OH by the MB‐fading reaction (Figure 2i). The FeRu nanozymes exerted excellent POD‐like activity under weakly acidic conditions (Figure 2j). Within the initial five minutes, the absorbance of MB significantly decreased, indicating the rapid generation of ·OH and its sustainability for an extended period. Subsequently, we evaluated the enzymatic kinetics of FeRu nanozymes using 3,3′,5,5′‐tetramethylbenzidine (TMB) as an indicator. The TMB colorimetric reaction followed typical Michaelis‐Menten kinetics under weakly acidic conditions, with a *K_M_* value and maximum reaction rates (*V*_max_) of 5.308 mM and 4.301 × 10^−7^ M·s^−1^, respectively (Figure 2k,l). Table S2 summarizes the POD‐like catalysis kinetics parameters of natural enzymes and some similar nanozymes. It can be seen that FeRu nanozymes have a fast catalytic rate, but the affinity for the substrate needs to be improved.

Although the significant production of ·OH by FeRu nanozymes can disrupt the redox balance at the tumor site and induce oxidative stress, the high expression of reduced GSH in the TME helps to consume the ·OH produced by the enzyme to maintain the redox balance. Consequently, we investigated whether FeRu nanozymes exhibited GSHOx‐like activity against GSH to disrupt this balance. The reaction of 5,5′‐Dithiobis‐(2‐nitrobenzoic acid) (DTNB) with GSH produces thionitrobenzoic acid (TNB), which has a maximum absorption peak at approximately 412 nm and is used to assess GSH levels (Figure 2m). The absorbance of TNB decreased significantly with increasing FeRu nanozyme concentrations, indicating that GSH was catalyzed to the oxidized form GSSG through the GSHOx‐like activity of FeRu nanozymes (Figure 2n). Subsequently, we evaluated the GSHOx‐like enzymatic kinetics of FeRu nanozymes, and the results showed typical Michaelis‐Menten kinetics with a *K_M_* value and maximum reaction rates (*V*_max_) of 1.558 mM and 5.175 × 10^−8^ M·s^−1^, respectively (Figure 2o,p). Table S3 summarizes the GSHOx‐like catalysis kinetics parameters of natural enzymes and some similar nanozymes. It can be seen that compared with other enzymes, FeRu nanozymes catalyze the substrate at a slower rate but have a better affinity for the substrate. A quantitative analysis of GSH was also conducted to measure the amount of GSH consumed (Figure S8). The enzyme kinetic results further elaborated the dynamic interaction between CAT‐like and POD‐like activities. The POD‐like activity has a much higher affinity for H_2_O_2_ (*K_m_* = 5.3 mM) than that of CAT‐like activity (*K_m_* = 135.8 mM). In the TME, POD is dominant, efficiently consuming H_2_O_2_ to generate •OH that immediately kills tumor cells. Although CAT‐like activity has a relatively lower affinity, it can continuously produce O_2_ and alleviate hypoxia. This creates a synergistic cycle: the O_2_ produced by CAT‐like activity can provide fuel for tumor metabolism to produce more H_2_O_2_, and then the POD pathway preferentially uses H_2_O_2_ to amplify oxidative stress.

In summary, we can speculate that FeRu nanozymes are a type of nanozyme with simultaneous CAT‐like, POD‐like, and GSHOx‐like activities. FeRu nanozymes quickly decompose H_2_O_2_ into O_2_ and ·OH. In the case of GSH, FeRu nanozymes consume GSH slightly more slowly but persistently.

### 2.3 DFT Simulation

**FIGURE 3:**
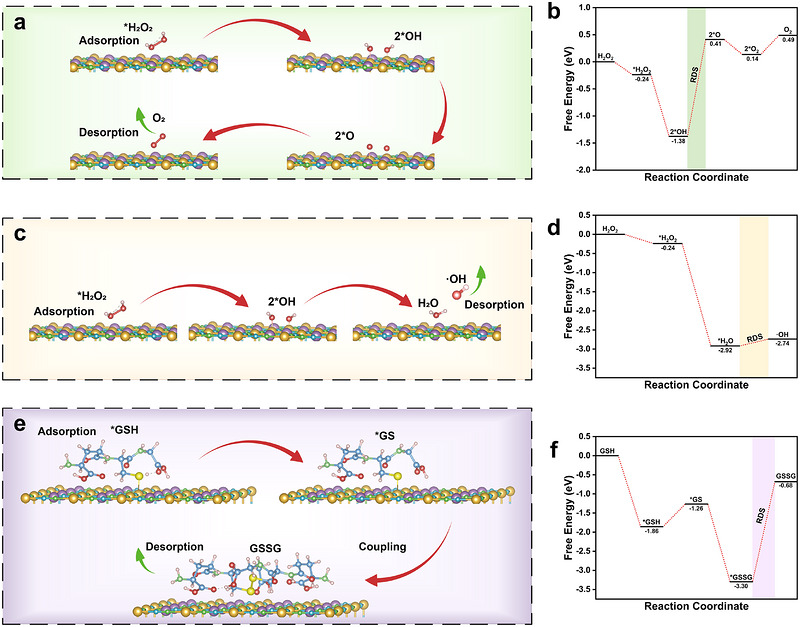
DFT simulation. The proposed catalytic mechanism of FeRu nanozymes for (a) CAT‐like reaction, (c) POD‐like reaction, and (e) GSHOx‐like reaction under simulated conditions of 37°C and pH 6.5. Free energy diagram of FeRu nanozymes for (b) CAT‐like reaction, (d) POD‐like reaction, and (e) GSHOx‐like reaction.

In order to analyze the potential catalytic mechanism of FeRu nanozymes, we provide more powerful data support for the three‐enzyme catalytic capabilities through density functional theory (DFT) calculation at the atomic level. In the CAT‐like reaction, H_2_O_2_ molecules are first adsorbed onto the surface of the nanozymes, where they dissociate into two *OH radicals. These radicals are subsequently dehydrogenated to produce two *O radicals, which then combine to form O_2_ before being released into the TME (Figure 3a). Analysis of the computational results indicates that the rate‐determining step (RDS) for the FeRu nanozymes in the CAT‐like reaction is the formation of *O following dehydrogenation, with a maximum free energy change (Δ*G*_max_) of 1.79 eV during this step (Figure 3b). Figure 3c shows the POD‐like catalytic process of the nanozymes. In POD‐like reactions, H_2_O_2_ molecules are similarly adsorbed onto the surface of the nanozymes and activated, producing an activated ·OH and *OH attached to the surface. In a weakly acidic environment, the protonated hydrogen atoms attached to *OH react to form water molecules, while simultaneously desorbing from the surface of the nanozymes. In this process, the Gibbs free energy decreases rapidly by −2.5 eV (Figure 3d). In POD‐like reactions, the desorption of *OH is the RDS, with the maximum free energy change (Δ*G*_max_) occurring during this step. Figure 3e shows the GSHOx‐like catalytic reaction process. The conformationally optimized GSH is adsorbed onto the surface of the nanozymes, becoming activated GSH (*GSH). The *GSH is then dehydrogenated to produce *GS, which subsequently forms *GSSG through coupling. Finally, the desorption of GSSG occurs, resulting in a significant decrease in Gibbs free energy of −0.68 eV. The RDS in the GSHOx‐like reaction of the nanozymes is the desorption of GSSG (Figure 3f). According to the above DFT calculations, the FeRu nanozymes demonstrated remarkable biomimetic activity in CAT‐like, POD‐like, and GSHOx‐like reactions, as indicated by the Δ*G*_max_ values.

The DFT‐calculated energy barriers for the rate‐determining steps provide a fundamental explanation for the Michaelis‐Menten kinetics we measured. For the CAT‐like reaction, the relatively high energy barrier of 1.79 eV for *O formation correlates well with the experimentally observed high Michaelis constant (*K_m_* = 135.84 mM), indicating a lower substrate affinity. On the contrary, for the POD‐like reaction, the overall lower energy pathway facilitates a faster reaction rate, which is consistent with the high maximum reaction velocity we measured (*V*_max_ = 4.301 × 10^−^^7^ M·s^−^^1^). Most notably, the GSHOx‐like reaction has the lowest energy barrier (1.58 eV) among the three reactions, which aligns perfectly with its optimal substrate affinity, with a minimum *K_m_* value of 1.558 mM. These clear correspondences demonstrate that our DFT model has successfully predicted the qualitative trends of FeRu enzyme kinetics.

Regarding temperature and pH values in the simulation calculation, the temperature of 37°C was incorporated through thermodynamic corrections to define the reaction rates and feasibility of the energy barriers (Δ*G*_max_) we reported. Crucially, the pH value of 6.5 was explicitly simulated in the reaction pathways: it provided the necessary protons (H^+^), significantly lowered the energy barrier for the rate‐determining step of *OH desorption in the POD‐like reaction, and ensured the correct protonation state of the GSH substrate to achieve GSHOx‐like activity. Therefore, the results of our DFT calculations can basically correspond to the TME in vivo.

### 2.4 Evaluation of Cytotoxicity In Vitro

In view of the strong three‐enzyme catalysis of FeRu nanozymes in vitro, as a premise, we first investigated the cytotoxicity of FeRu nanozymes in B16 cells. In the experimental treatments, both CAT and BSO were included, respectively, to effectively reduce the catalytic substrates H_2_O_2_ and GSH. As mechanism‐probing controls, they help to elucidate the tumor cell damage mechanism of FeRu nanozymes from the perspective of multi‐enzyme catalysis.

**FIGURE 4:**
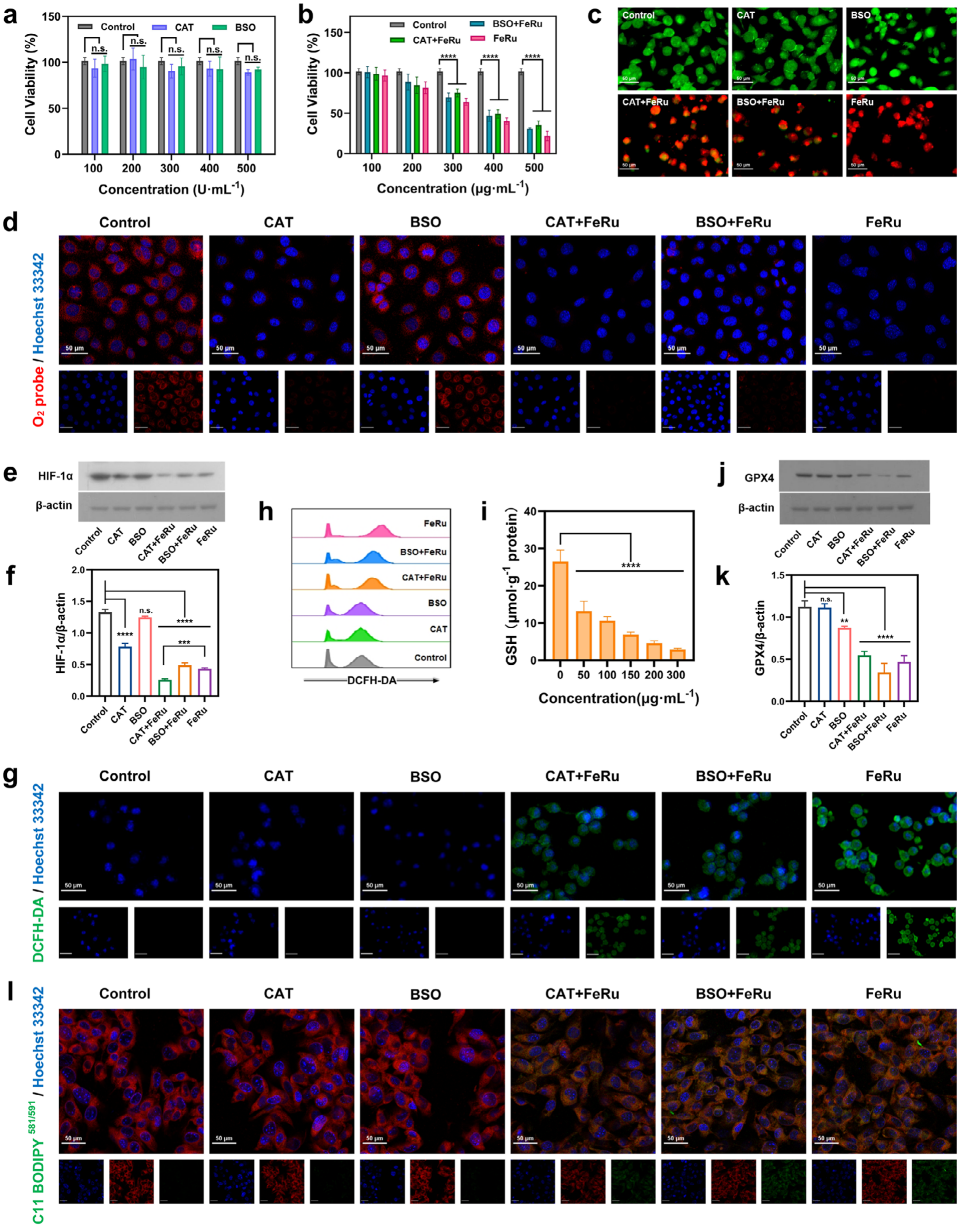
Evaluation of multi‐enzyme activity and related protein expression in vitro. (a,b) Cytotoxicity profiles of B16 cells after different treatments. (c) The fluorescent images of B16 cells after different treatments, stained with calcein‐AM (green signal) and PI (red signal), respectively. (d) Fluorescent images of B16 cells in various groups and stained with O_2_ probe. (e) Western blot analysis of HIF‐1α expression levels and (f) relative content of B16 cells treated with different formulations. (g) Fluorescent images of DCFH‐DA assays of B16 cells incubated with different treatments. (h) Flow cytometry analysis of ·OH generation in B16 cells after different treatments. (i) Intracellular GSH levels of B16 cells after incubation with various concentrations of FeRu nanozymes. (j) Western blot analysis of GPX4 expression levels and (k) relative content of B16 cells treated with different formulations. (l) Fluorescent images of B16 cells in various groups and stained with BODIPY^TM^ 581/591 C^11^ (*n* = 3, **p < 0.01, ****p < 0.0001, ns, no significant).

The survival rate of cells incubated with CAT or BSO alone was comparable to that of the control group (Figure 4a), suggesting that the concentration selection of these two inhibitors is appropriate. As shown in Figure 4b, the cytotoxicity of the cells treated with FeRu nanozymes was slightly higher than that of the other groups. CAT effectively consumed most of the H_2_O_2_ in the cells, breaking it down into O_2_. Consequently, the H_2_O_2_ content catalyzed by the POD‐like activity of FeRu nanozymes was significantly reduced, which restricted the production of ·OH. Additionally, BSO decreased the intracellular GSH content to some extent. Therefore, after co‐incubation with CAT or BSO, the level of oxidative stress in cells was reduced, leading to relatively higher cell vitality, which further proved that FeRu nanozymes possess three catalytic properties: CAT‐like, POD‐like, and GSHOx‐like activities. Moreover, calcein‐acetoxymethyl ester (AM) and propidium iodide (PI) were used to stain the living and dead cells for visualization under a fluorescence microscope (Figure 4c). These results indicated that FeRu nanozymes have an excellent tumor‐killing effect. Furthermore, additional MTT assay results demonstrated that the nanozyme exhibited significant antitumor activity in 4T1 cells (Figure S9) while showing no obvious cytotoxicity in L929 normal cells (Figure S10). These findings collectively verify the broad‐spectrum anticancer potential of the nanozymes and their negligible toxic effects on normal cells.

### 2.5 Evaluation of Intracellular Catalytic Products

We first measured O_2_ levels in B16 cells using an oxygen probe [Ru(dpp)_3_]Cl_2_ (Figure 4d). In the CAT group, a substantial amount of oxygen was generated to quench the red fluorescence of the probe. Similarly, abundant O_2_ was produced in the three groups containing FeRu nanozymes. The red fluorescence in these groups was severely quenched, indicating that FeRu nanozymes exhibited excellent CAT‐like activity to alleviate hypoxia in the TME. Subsequently, we further analyzed the expression of HIF‐1α after different treatments. As illustrated in Figure 4e,f, the expression of HIF‐1α was significantly reduced in the CAT group. Similarly, we observed a notable decrease in HIF‐1α expression in the three groups treated with FeRu nanozymes. Based on the above results, we concluded that FeRu nanozymes effectively exhibited CAT‐like activity in tumor cells, generating large amounts of oxygen.

Next, we investigated the ability of FeRu nanozymes to induce oxidative stress in vitro. 2′,7′‐Dichlorofluorescein diacetate was used as an ·OH fluorescent probe. The three groups containing FeRu nanozymes exhibited clear green fluorescence (Figure 4g). These results indicated that FeRu nanozymes could effectively generate ·OH in B16 cells, leading to oxidative stress. Flow cytometry (FCM) was also utilized to analyze intracellular ROS levels, and the results were consistent with the aforementioned findings (Figure 4h). Besides, the quantitative results clearly demonstrate a significant increase in fluorescence intensity in the FeRu nanozymes‐treated group compared to the control group (Figure S11).

We then evaluated the GSHOx‐like activity of FeRu nanozymes by quantitatively analyzing intracellular GSH levels, which gradually decreased with increases in FeRu nanozyme concentrations (Figure 4i). These results indicated that FeRu nanozymes could effectively consume intracellular GSH. Additionally, the overexpression of endogenous GSH in cancer cells can deplete ROS and activate GPX4 expression. Therefore, we examined the expression of GPX4 in cells after different treatments (Figure 4j,k). The results showed that the consumption of GSH by FeRu nanozymes significantly downregulated the expression of GPX4.

**FIGURE 5:**
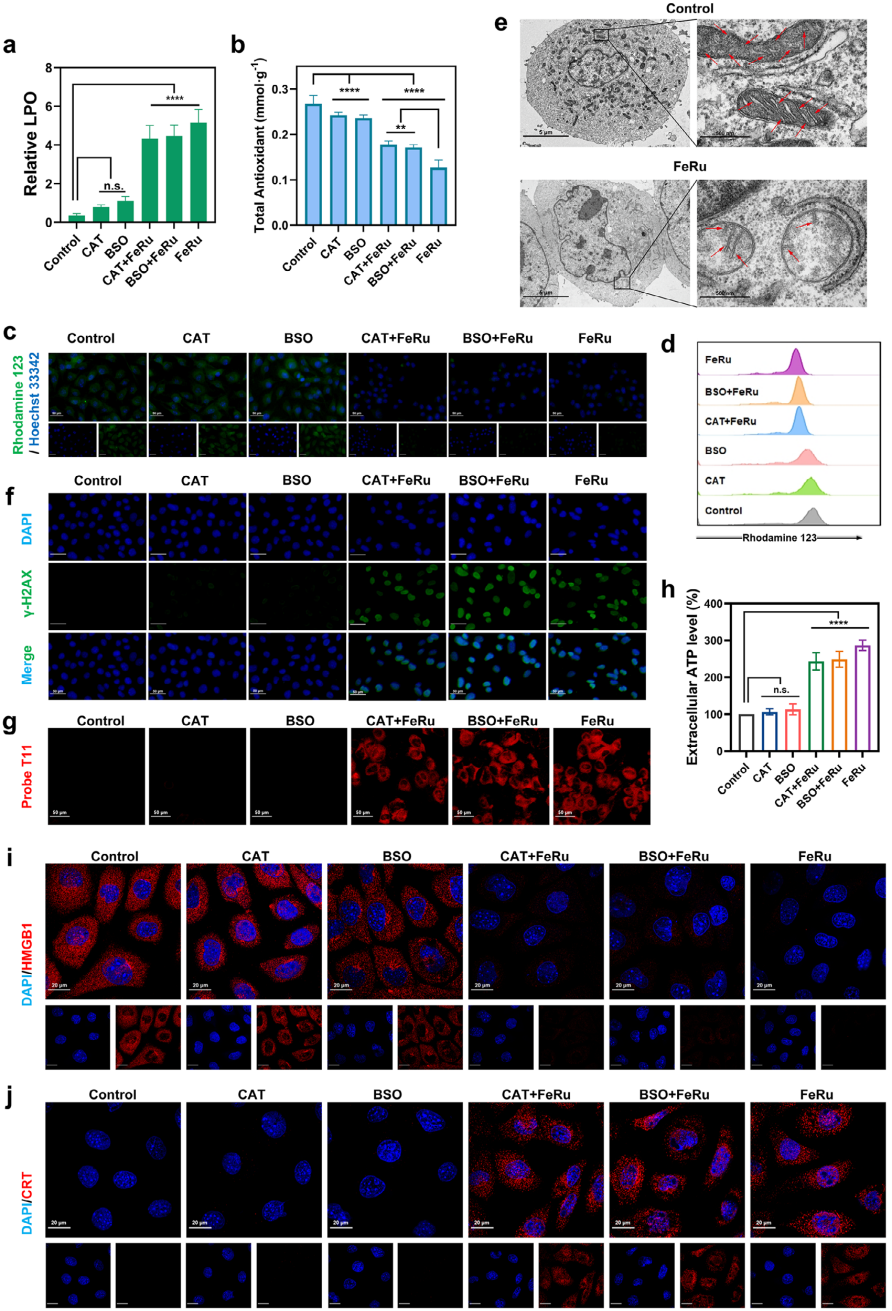
Evaluation of cell damage in vitro. (a) Relative LPO levels of B16 cells incubated with different treatments. (b) Total antioxidant levels of B16 cells incubated with different treatments. (c) Fluorescence images and (d) flow cytometry analysis of Rhodamine 123 assays of B16 cells incubated with different treatments. (e) Representative Bio‐TEM images of B16 cells in the control and FeRu nanozyme treatment groups. The red arrow represents the cristae of mitochondria. (f) Immunofluorescence image of B16 cells DNA damage in B16 cells under different treatments. (g) Fluorescence image of B16 cells membrane integrity of B16 under different treatments. (h) Extracellular ATP levels of B16 cells under different treatments. (i) Immunofluorescence images of HMGB1 in B16 cells with various treatments. (j) Immunofluorescence images of CRT in B16 cells with various treatments (*n* = 3, **p < 0.01, ***p < 0.001, ****p < 0.0001, ns, no significant).

When ROS accumulates in cells to a certain extent, it causes lipid peroxidation (LPO). The group treated with FeRu nanozymes exhibited strong green fluorescence, suggesting more pronounced LPO in the cells (Figure 4l). As expected, the relative LPO levels of the three groups treated with FeRu were significantly higher than those of the other three groups. In addition, the level of LPO in the cells was quantified, and the FeRu nanozymes group was the highest (Figure S12). The results of intracellular LPO were consistent with the previous trend in the breakdown of redox homeostasis (Figure 5a). Next, we investigated the total antioxidant capacity of the cells. An intracellular oxidative stress storm occurred with the accumulation of intracellular ROS, leading to a significant reduction in the total antioxidant capacity of the cells (Figure 5b).

During cellular oxidative stress, the loss of mitochondrial membrane potential resulted in a significant decrease in yellow‐green fluorescence intensity in the mitochondria. As expected, the fluorescence signals in the groups treated with FeRu nanozymes were notably decreased compared to the other three groups (Figure 5c). The results of the FCM analysis aligned with those of the fluorescence analysis (Figure 5d).

We further observed the morphological changes in B16 cells after treating them with FeRu nanozymes using Bio‐TEM. The results revealed that FeRu nanozymes could induce significant mitochondrial damage in cancer cells, which was characterized by morphological rounding, increased membrane density, and the reduction or loss of mitochondrial ridges. No notable cell retraction, chromatin aggregation, or apoptotic body formation was observed (Figure 5e). In conclusion, FeRu nanozymes could increase oxidative stress and trigger mitochondrial damage in tumor cells.

ROS can also cause DNA damage. Therefore, we studied DNA damage in the treated cells. Under normal culture conditions (control group), an almost invisible green signal was observed within the cells, as was the case in the CAT and BSO groups (Figure 5f). In contrast, the strong green fluorescence observed in all three FeRu nanozyme treatment groups indicated that phosphorylated γ‐H2AX was formed after DNA damage. The intense oxidative stress storm inside the cell caused by FeRu nanozymes destroyed the double‐stranded DNA.

In addition, in the initial stage of oxidative stress, the selective permeability of the cell membrane has been proven to be impaired. The results showed that the cells in the three groups containing FeRu nanozymes exhibited significant red fluorescence, indicating impaired membrane integrity (Figure 5g), which is consistent with the results of quantitative analysis (Figure S13). We inferred that the accumulation of ROS and subsequent LPO induced by FeRu nanozymes led to a gradual decrease in membrane phospholipids.

FeRu nanozymes not only possess three distinct enzyme‐like activities but also integrate them into a self‐reinforcing catalytic cycle to systematically reshape the TME. The CAT‐like activity initiates the therapeutic cascade by decomposing intratumoral H_2_O_2_ to produce O_2_. This not only directly alleviates hypoxia but also provides a substrate for the regeneration of H_2_O_2_ in tumor metabolism. This oxygen‐replenished and H_2_O_2_‐enriched microenvironment, in turn, strongly enhances the POD‐like activity, converting H_2_O_2_ into highly toxic •OH and triggering a destructive oxidative stress storm. Simultaneously, the GSHOx‐like activity depletes the key antioxidant GSH, thereby severely disrupting the main defense system in tumors, reducing the clearance rate of •OH, and allowing oxidative damage to spread unimpeded. These three activities operate in a positive feedback mode, with the output of one reaction providing fuel or promoting the next, thus forming a comprehensive TME remodeling state; the efficacy far exceeds that of nanozymes with single or double activities.

### 2.6 Activation of Cellular Immunity In Vitro

In addition to the excellent multi‐enzyme activity of FeRu nanozymes in cells, we also intended to explore whether FeRu nanozymes could initiate immune responses in tumors, thereby remodeling the tumor immunosuppressive microenvironment. We found that B16 cells treated with FeRu nanozymes showed a significantly enhanced release of DAMPs. The cells treated with FeRu nanozymes released a large amount of ATP, as reflected by elevated ATP content in the cell culture medium (Figure 5h). Additionally, high mobility group box‐1 protein (HMGB1) was highly expressed in cells that had not been treated with FeRu nanozymes, and obvious red fluorescence was observed by confocal laser scanning microscope (Figure 5i), and the mean fluorescence intensity (MFI) is shown in Figure S14. In contrast, the red fluorescence signals disappeared in cells treated with FeRu nanozymes, suggesting that they released a large amount of HMGB1 into the extracellular area. Meanwhile, compared with the other groups, cells treated with FeRu nanozymes exhibited a large amount of intense red fluorescence (Figure 5j), showing that the intense oxidative stress significantly induced CRT exposure to the surface of tumor cells. The MFI is shown in Figure S15.

### 2.7 The Anti‐Tumor Efficacy of Different Administration Frequencies

**FIGURE 6:**
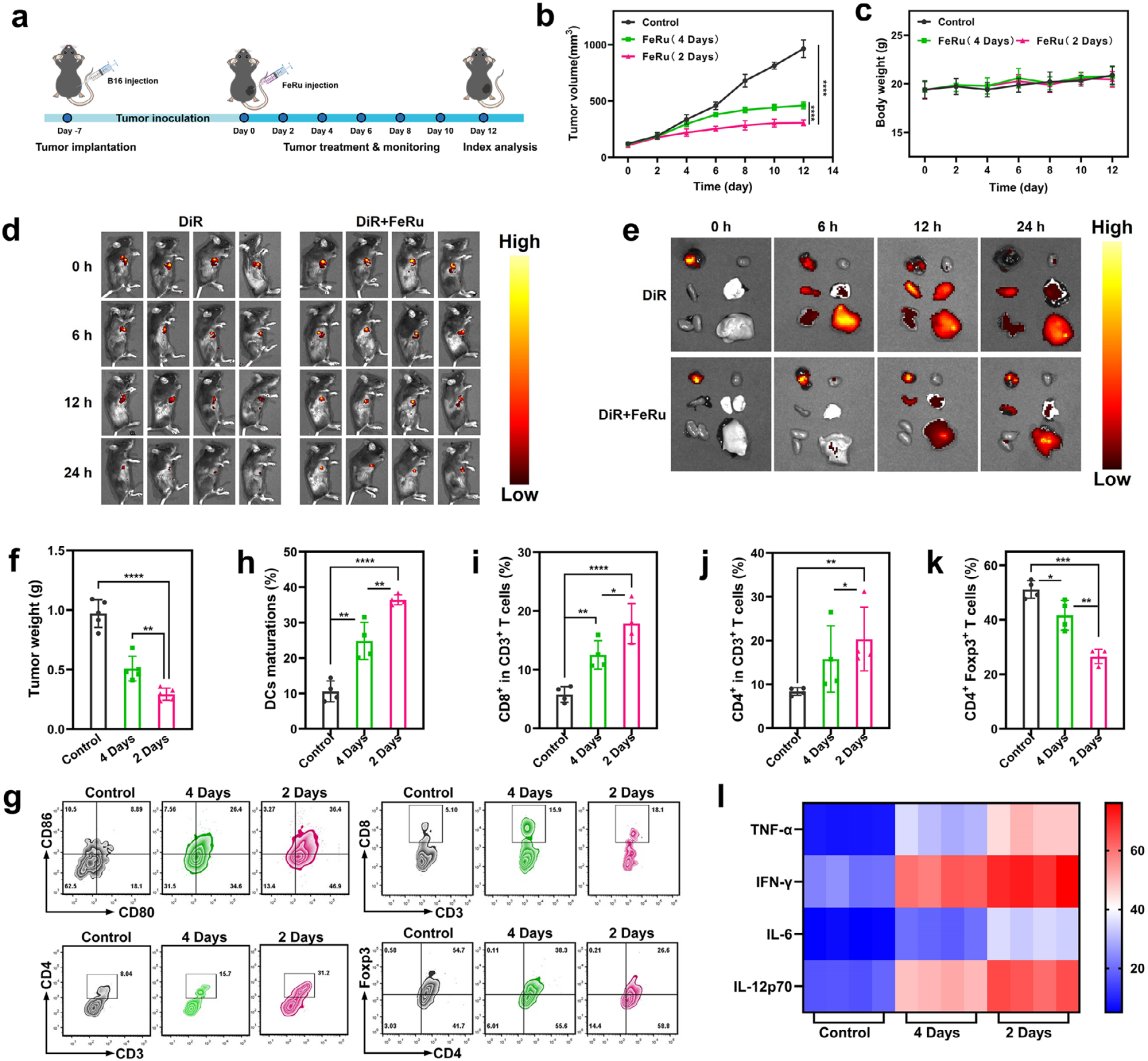
Evaluation of in vitro anti‐tumor properties of FeRu nanozymes in different treatment frequencies. (a) Schematic treatment schedule. (b) Tumor growth curves and (c) body weight changes and (f) ex vivo tumor weight of mice with various treatments. (d) In vivo fluorescence images of tumors at various time points after intratumoral injection of various agents. (e) Ex vivo biodistribution in major organs at various time points after intratumoral injection of various agents. (g) Representative FCM profiles of DCs, CD8^+^T cells, CD4^+^T cells, and T_regs_ in the tumors with various treatments. The percentages of (h) DCs, (i) CD8^+^T cells, (j) CD4^+^T cells, and (k) T_regs_ populations in tumors with various treatments. (l) The heat map of intratumoral levels of TNF‐α, IFN‐γ, IL‐6, and IL‐12 after different treatments (b–f: *n* = 5, *p < 0.05, **p < 0.01, ***p < 0.001, ****p < 0.0001; d,e,h–l: *n* = 4, *p < 0.05, **p < 0.01, ***p < 0.001, ****p < 0.0001).

First, we investigated the difference in the therapeutic effect of different administration frequencies on tumors in mice (Figure 6a). During the 12‐day observation period, the tumor growth rate of mice in the control group was particularly fast, while the tumor inhibition effect was significant in the FeRu nanozyme groups, and the effect was better in the group treated every 2 days (Figure 6b and Figure S16). The weights of the mice in different groups were slightly different but were not statistically different (Figure 6c). These results indicated that FeRu nanozymes could effectively inhibit tumor progression, especially when administered every 2 days. Furthermore, the in vivo imaging and tumor accumulation results (Figure 6d) indicated that the nanozyme exhibited peak fluorescence intensity at the tumor site 12 h after injection, and the signals persisted for over 24 h, which was significantly different from the rapid clearance observed with free DiR. This “slow accumulation–prolonged retention” feature is in line with the 2‐day dosing regimen and can ensure continuous drug coverage. The findings of tissue distribution further support the long‐acting properties of the nanozyme and the high accumulation in tumor sites (Figure 6e and Figure S17). The average tumor weight in the 2‐day group was only 0.29 g, which was 29.6% of that in the control group and 56.9% of that in the 4‐day group (Figure 6f).

First, the maturation of dendritic cells (DCs) in tumors was studied using FCM. A representative flow cytometric analysis of DCs, CD8^+^T cells, CD4^+^T cells, and T_reg_ cells is shown in Figure 6g. The results in Figure 6h revealed that the percentage of mature DC cells in mice treated for 4 days was about 2.2 times higher than that in the control group (24.8% vs. 11.3%). The percentage of mature DC cells in mice in the 2‐day group was approximately 3.2 times higher than that of mice in the control group (36.4% vs. 11.3%). This suggests that FeRu nanozymes could effectively promote DC maturation, and increasing the administration frequency was found to be more effective. Studies have indicated that mature DC cells can present antigens to T cells, thereby activating T cells at the tumor site and initiating an immune response [46]. The results suggested that tumors from FeRu nanozymes‐treated mice contained significantly higher percentages of CD8^+^ T cells and CD4^+^ T cells than the control group (Figure 6i,j). We further studied the immunosuppression of regulatory T (T_reg_) cells in different groups. The percentage of T_reg_ cells in the tumors from FeRu nanozymes‐treated mice was significantly lower than that in other groups (Figure 6k). In addition, tumor necrosis factor‐α (TNF‐α), interferon‐gamma (IFN‐γ), IL‐6, and IL‐12p70 were significantly upregulated after FeRu nanozymes administration, confirming that FeRu nanozymes remodel the TME most effectively in the 2‐day group (Figure 6l).

The cytotoxicity of CD8^+^ T cells represents a direct antitumor mechanism. The significant increase in tumor‐infiltrating CD8^+^ T cells indicates successful activation of the cancer‐immunity cycle. FeRu nanozymes‐induced immunogenic cell death releases tumor antigens, which are captured by DCs, leading to the priming and activation of tumor‐specific CD8^+^ T cells in lymph nodes. These cytotoxic T lymphocytes subsequently infiltrate the tumor, mediating tumor cell killing – this is the primary effector mechanism underlying the observed tumor suppression [47]. Moreover, the reduction in T_regs_ is key to relieving immunosuppression. T_regs_ typically suppress effector T cell function through various mechanisms, including cytokine secretion and metabolic disruption. Their reduction directly alleviates this immunosuppressive pressure, thereby enhancing the cytotoxic activity of CD8^+^ T cells. The improved CD8^+^/T_reg_ ratio is a recognized indicator of effective antitumor immunity and directly contributes to the therapeutic outcome. Besides, the pro‐inflammatory microenvironment produced by the nanozyme (characterized by elevated IFN‐γ levels) can induce the upregulation of PD‐L1 on tumor cells [48], which lays the foundation for the subsequent combined study with αPD‐L1 antibodies in this research.

### 2.8 The Anti‐Tumor Effect of Different Administration Groups

**FIGURE 7:**
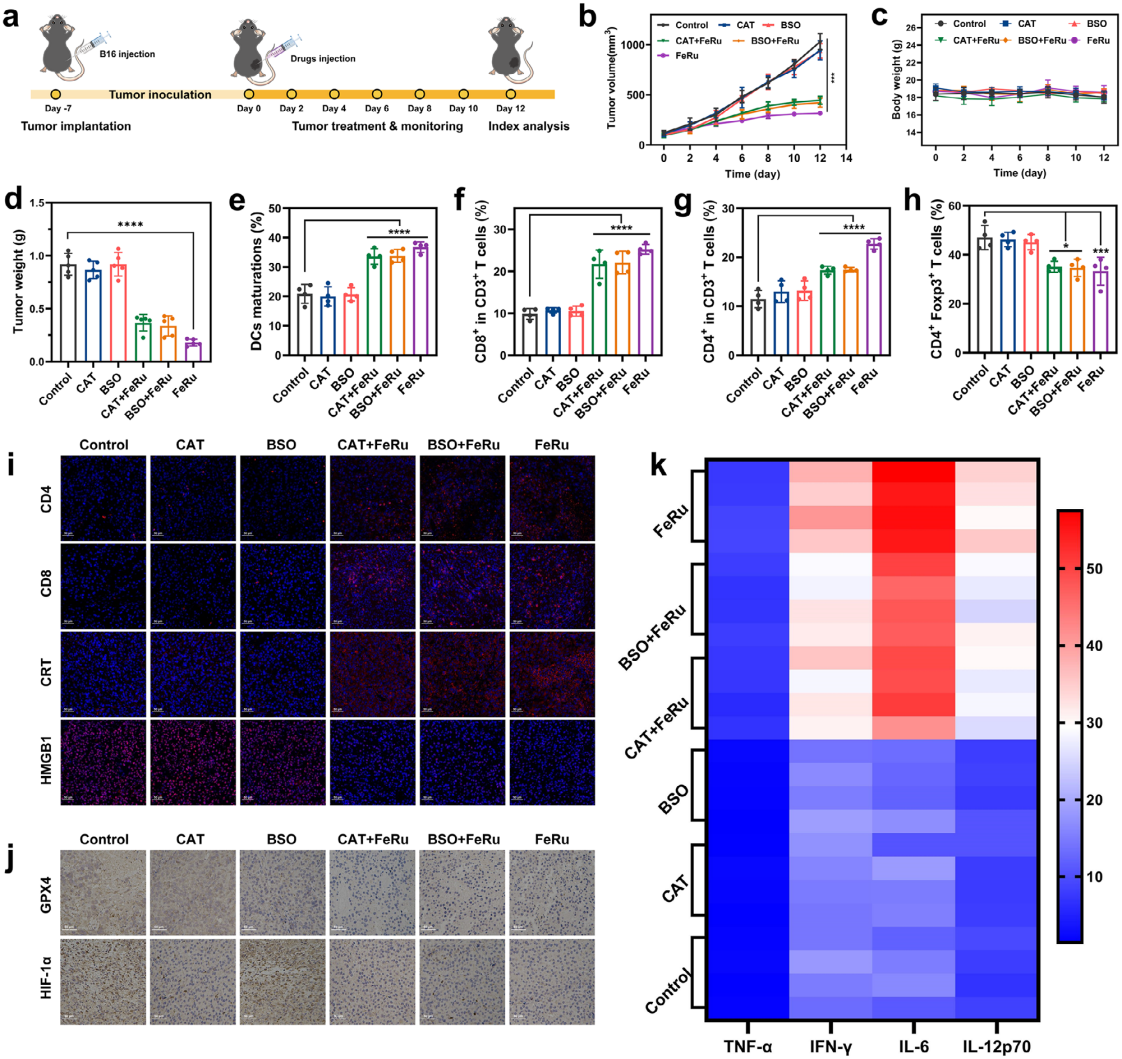
Evaluation of in vitro anti‐tumor properties of FeRu nanozymes in different treatment combinations. (a) Schematic treatment schedule. (b) Tumor growth curves and (c) body weight changes and (d) ex vivo tumor weight of mice with various treatments. The percentages of (e) DCs, (f) CD8^+^T cells, (g) CD4^+^T cells, and (h) T_regs_ populations in tumors with various treatments. (i) Immunofluorescence staining of CD4^+^T cells, CD8^+^T cells, CRT, and HMGB1 expression in the tumor tissues. (j) Histological images of the KHOS‐bearing mice tumor tissues of KHOS‐bearing mice with various treatments stained by GPX4 and HIF‐1α. (k) The heat map of intratumoral levels of TNF‐α, IFN‐γ, IL‐6, and IL‐12 after different treatments. (b–d: *n* = 5, *p < 0.05, **p < 0.01, ***p < 0.001, ****p < 0.0001; e–h,j: *n* = 4, *p < 0.05, **p < 0.01, ***p < 0.001, ****p < 0.0001).

With reference to the above experimental results, the subsequent administration frequency of FeRu nanozymes was set to once every 2 days (Figure 7a). The results during the 12‐day treatment period indicated that the group treated with FeRu nanozymes exhibited significant tumor growth inhibition (Figure 7b and Figure S18). Besides, no statistically significant difference in mouse weights was seen in the different treatment groups (Figure 7c). Furthermore, the average tumor weight of mice in the FeRu nanozymes treatment group was only 0.18 g, which accounted for 19.78% of the control group (Figure 7d).

Immune profiling showed a notable increase in the proportion of mature DCs, CD8^+^ T, and CD4^+^ T cells alongside a decrease in the levels of T_reg_ cells in the three groups treated with FeRu nanozymes (Figure 7e–h and Figure S19). This demonstrated that FeRu nanozymes exhibited significant immune activation. Immunofluorescence confirmed enhanced CD4/CD8 infiltration and CRT exposure, with diminished HMGB1 fluorescence indicating extracellular release in the groups treated with FeRu nanozymes (Figure 7i). H&E and terminal deoxynucleotidyl transferase uridine triphosphate nick end labeling (TUNEL) staining revealed extensive tumor cell death, and Ki67 staining showed proliferation inhibition (Figure S20). Immunohistochemical analysis showed that the expression of GPX4 and HIF‐1α was significantly downregulated in the FeRu nanozymes‐treated group (Figure 7j), indicating increased oxidative stress levels in the tumors. In addition, TNF‐α, IFN‐γ, IL‐6, and IL‐12p70 were significantly upregulated after FeRu nanozyme administration (Figure 7k).

### 2.9 The Anti‐Tumor Effect of FeRu Nanozymes Combined With αPD‐L1

Through multidimensional comparisons with conventional antibody drugs and cell therapies, the nanozyme platform developed in this study demonstrates distinctive advantages. First, compared with traditional antibodies, the high stability of nanozymes enables them to withstand the influence of temperature, pH value, and chemical denaturants, maintain their activity in body fluid environments, and do not require cold chain transportation, significantly reducing application costs. In contrast, traditional antibodies are prone to inactivation under the influence of high temperatures or chemical denaturants, and they have strict requirements for storage and transportation conditions. Second, nanozymes can simultaneously simulate the activities of multiple natural enzymes (such as CAT and POD) and generate synergistic effects through cascade reactions. Traditional antibodies only exert their effects through a single target and cannot achieve multi‐mechanism synergy. Moreover, the catalytic reaction of nanozymes can also release tumor‐associated antigens, activate DCs to present antigens, and regulate the immune microenvironment through multiple pathways, initiating a systemic anti‐tumor immune response, with more lasting therapeutic effects. However, traditional antibody drugs (such as ICIs) may cause disruption of immune balance or silencing of immune responses. Meanwhile, compared with the currently popular cell therapy, nanozymes do not require strict cold chain transportation (below −80°C) and complex in vitro amplification and genetic modification, reducing the high treatment cost. Moreover, nanozymes alleviate tumor hypoxia through catalytic reactions, consume GSH, and reverse the immunosuppressive state. However, the efficacy of cell therapy in the microenvironment of solid tumors (such as hypoxia and infiltration of immunosuppressive cells) will be weakened. For example, CAR‐NK cells are prone to being induced into an exhausted state, resulting in impaired function.

**FIGURE 8:**
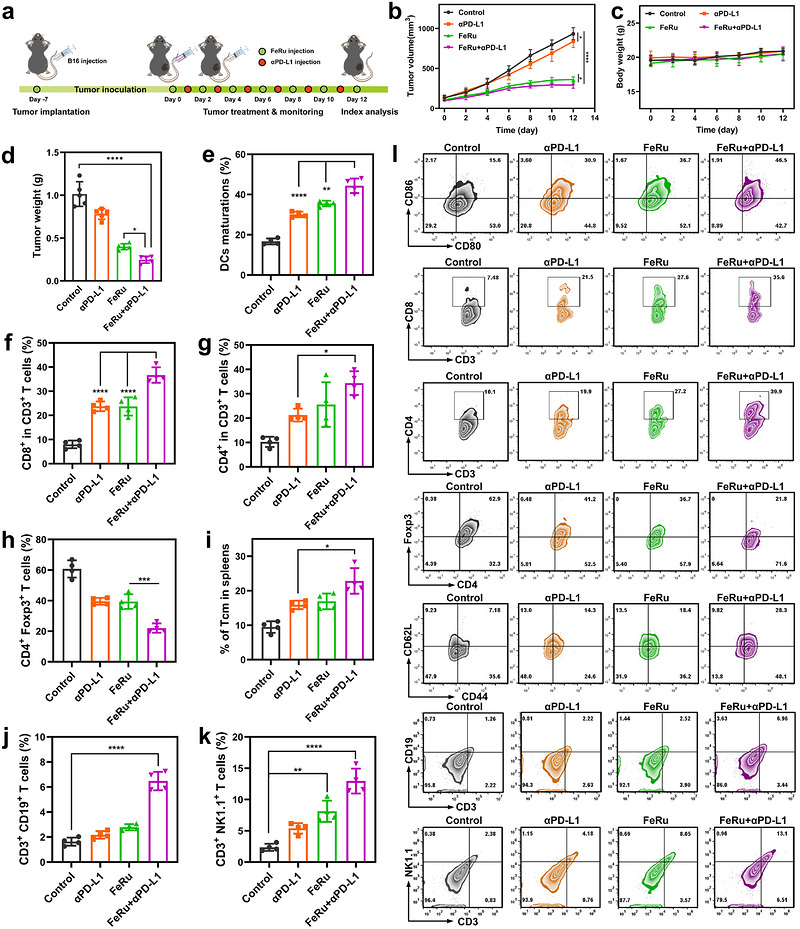
Evaluation of in vitro anti‐tumor properties of FeRu nanozymes in combination with α PD‐L1. (a) Schematic treatment schedule. (b) Tumor growth curves and (c) body weight changes and (d) ex vivo tumor weight of mice with various treatments. The percentages of (e) DCs, (f) CD8^+^T cells, (g) CD4^+^T cells, (h) T_reg_ *_s_*, (i) *T*_cm_, (j) B cells, and (l) NK cell populations in tumors with various treatments. (l) Representative FCM profiles of DCs, CD8^+^T cells, CD4^+^T cells, T_regs_, T_cm_, B cells, and NK cells in the tumors with various treatments (b–d: *n* = 5, *p < 0.05, **p < 0.01, ***p < 0.001, ****p < 0.0001; e–k: *n* = 4, *p < 0.05, **p < 0.01, ***p < 0.001, ****p < 0.0001).

Building on these strengths, we further investigated the synergistic effects of combining FeRu nanozymes with αPD‐L1 antibody (Figure 8a). Both the FeRu nanozymes and αPD‐L1 + FeRu nanozymes groups showed significant tumor suppressive effects, especially the αPD‐L1 + FeRu nanozymes group (Figure 8b–d and Figure S30), with no significant change in the body weight of the mice (Figure 8c).

Immune profiling showed a notable increase in the proportion of mature DCs, CD8^+^ T cells, and CD4^+^ T cells alongside a decrease in the levels of T_reg_ cells in the combination group (Figure 8e–h), while the number of central memory T cells (T_cm_) in the spleen of this group increased, approximately 2.4 times higher than the control group (22.8% vs. 9.4%) (Figure 8i). In addition, compared with the control group, the numbers of NK cells and B cells in the FeRu alone and FeRu + αPD‐L1 combination treatment groups significantly increased, especially for NK cells (Figure 8j,k). The representative flow cytometric analysis of mature DCs, CD8^+^ T cells, CD4^+^ T cells, T_regs_, T_cm_, B cells, and NK cells is shown in Figure 8l. Moreover, the cytokines (TNF‐α, IFN‐γ, IL‐6, and IL‐12p70) in the combination group were significantly increased (Figure S22). This indicates that the nanozyme can not only activate adaptive immunity but also innate immunity. NK cells inhibit tumors through direct cytotoxicity and IFN‐γ secretion. This result provides a more comprehensive view of the powerful immune activation achieved by FeRu nanozymes. The above‐mentioned synergistic effect effectively reversed TME immunosuppression and amplified anti‐tumor immunity (gating strategies: Figure S23–S29).

In summary, our catalytic immunomodulation strategy differs from other immunotherapy modalities. Unlike cell therapies, which often require complex ex vivo manipulation and face challenges infiltrating solid tumors, our approach enables the in situ generation and expansion of tumor‐specific T cells within the TME. Compared to αPD‐L1 monotherapy, which often fails in immunologically “cold” tumors, the FeRu nanozymes effectively convert the TME into an immunologically “hot” state, creating favorable conditions for checkpoint blockade therapy. This positions our strategy as an effective method to sensitize tumors to existing immunotherapies.

### 2.10 Biosafety of FeRu Nanozymes

**FIGURE 9:**
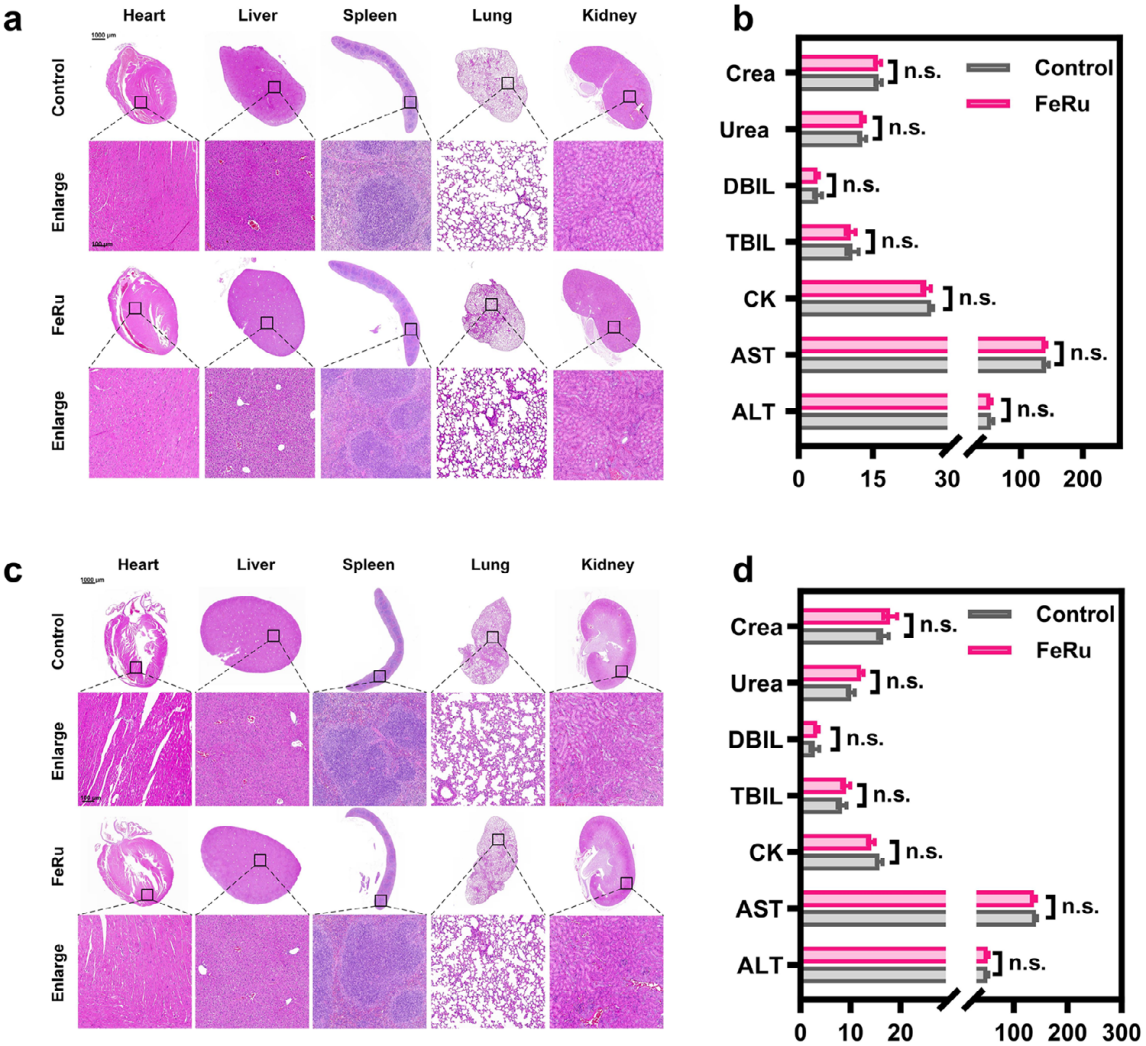
In vivo biosafety assessment of the FeRu nanozymes. H&E‐stained sections of major organs (heart, liver, spleen, lung, and kidney) from mice after (a) 30 days and (c) 45 days of administration of nanozymes. Serum biochemical analysis at corresponding time points: levels of main biochemical indicators after (b) 30 days and (d) 45 days. The assessed parameters are ALT (U/L), AST (U/L), CK × 100 (U/L), TBIL (µmol/L), DBIL (µmol/L), urea (mmol/L), and creatinine (µmol/L) (*n* = 3, ns, no significant difference).

We systematically evaluated the short‐term and long‐term biosafety and biocompatibility of FeRu nanozymes in mice at 12 days, 30 days, and 45 days after administration. H&E staining of major organs (heart, liver, spleen, lungs, and kidneys) further confirmed histological integrity with no observed damage (Figure S30 and Figure 9a–c). Hematological and biochemical parameters showed no significant abnormalities in function markers (serum alanine transaminase, aspartate aminotransferase, total bilirubin, direct bilirubin, ureaphil, creatinine, and creatine kinase) compared with the control group (Figure S31, Figure 9b–d). Overall, these results demonstrate the favorable long‐term biosafety profile of the nanozyme.

## 3 Conclusion

In summary, we synthesized a ruthenium‐doped hollow iron‐based nanozyme for efficient malignant tumor therapy. FeRu nanozymes possess zero‐valent metal electron transport bridges, large specific surface areas, and abundant metal catalytic active sites. It not only possesses three distinct enzyme‐like activities but also integrates them into a self‐reinforcing catalytic cycle to systematically reshape the TME, with efficacy far exceeding that of single‐enzyme or dual‐enzyme activity nanozymes. These processes activate the adaptive and innate immune response and have shown excellent therapeutic effects in the combined application of αPD‐L1. In addition, this nanozyme has demonstrated good long‐term safety, which implies a great possibility of clinical application and provides a good reference for the research of other nanozymes. On this basis, future work will focus on how to further enhance the catalytic performance of nanozymes and increase their affinity for substrates, such as achieving functionalization through surface engineering (introducing targeted molecules or specific amino acids), doping heteroatoms to regulate the electronic structure, or optimizing the pore structure to enhance mass transfer. These methods are expected to provide new approaches for the design of highly efficient nanozymes.

## Ethics Statements

All animal protocols were granted approval by the Animal Research Committee of Shenyang Pharmaceutical University (No: SYPU‐IACUC‐S2025‐0430‐105).

## Conflicts of Interest

The authors declare no conflicts of interests.

## Supporting information




**Supporting File**: exp270209‐sup‐0001‐SuppMat.docx.

## Data Availability

All data supporting the funding of this study are available from the corresponding authors upon reasonable requests.
