# Supplementary material for: Zero‐Valent Ruthenium‐Anchored Nanozymes Remodel Tumor Microenvironment for Malignant Tumor Treatment in Combination With αPD‐L1 Immunotherapy
Source: Exploration (Beijing). 2026 Aug 10;6(4):70209. doi: 10.1002/exp2.70209 (PMC13457402; doi:10.1002/exp2.70209)
Supplement: Supplementary file 1 [file EXP2-6-70209-s001.docx]

**Supporting Information**

**Zero-valent Ruthenium-anchored Nanozymes Remodel Tumor Microenvironment for Malignant Tumor Treatment in Combination with αPD-L1 Immunotherapy**

**Experimental section**

**Materials**

Albumin Bovine (BSA) was obtained from Biosharp. (Hefei, China). Ruthenium(Ⅲ) chloride trihydrate (RuCl_3_·3H_2_O), methylene blue (MB), tetramethylbenzidine (TMB), 5,5′Dithiobis-(2-nitrobenzoic acid) (DTNB), glutathione (GSH), potassium Hexacyanoferrate (K_3_[Fe(CN)_6_]) and PVP were bought from Macklin Co. (Shanghai, China). Tris, DMEM medium, fetal bovine serum (FBS), RPMI 1640 medium, BCA protein assay kit, MTT cell proliferation, cytotoxicity assay kit, Annexin V-FITC/ PI apoptosis detection kit, Calcein AM/PI cell viability assay kit, DCFH-DA and DiR iodide [1,1’-dioctadecyl-3,3,3’,3’-tetramethylindotricarbocyanine iodide] were also acquired from Meilun Biotechnology Co. (Dalian, China). DNA damage assay kit by γ-H2AX immunofluorescence, lipid peroxidation assay kit, total antioxidant capacity assay kit, GSH and GSSG assay kit, rhodamine 123, autophagy staining assay kit with MDC, Lyso-Tracker red, HIF-1α rabbit polyclonal antibody, Alexa Fluor 555-labeled Donkey Anti-Rabbit IgG (H+L), DAPI, HMGB1, CRT, were purchased from the Beyotime Biotechnology Co. (Haimen, China). C11-BODIPY^581/591^ lipid peroxidation sensor was obtained from Maokang Biotechnology Co. (Shanghai, China). Ammonium Bis (Oxalato) Oxotitanate (IV) Hydrate were purchased from Acmec Biochemical Co. Ltd. (Shanghai, China). Anti-GPX4 polyclonal antibody was purchased from Solarbio Co. (Beijing, China). Anti-mouse PD-L1 was obtained from Bio X Cell Co. (American).

**Characterization**

The morphology and structure of FeRu nanozymes was observed using transmission electron microscopy (TEM, Tecnai G20, FEI, USA). The hydrodynamic diameter and zeta potential were measured using the Malvern Zetasizer Nano series (Malvern, UK). The chemical composition was evaluated by X-ray photoelectron spectroscopy (XPS, ESCAlab250, Thermo Fisher Scientific, USA). The crystallization state analyzed by X-ray diffraction (D8 Advance, Bruker, Germany). Localization imagings of all the fluorescence slides were performed using a confocal laser scanning microscope (CLSM) (Nikon, Japan) and a fluorescence microscope (Mshot, Guangzhou, China).

**Synthesis of FeRu nanozymes**

The hollow cubic-like FeRu nanozymes were synthesized by a facile one-pot hydrothermal method. In a typical synthesis, PVP (3 g) were dissolved in HCl (40 mL) solution under stirring. Then, RuCl_3_·3H_2_O (51.85 mg, 0.25 mmol) and K_3_[Fe(CN)_6_] (82.31 mg, 0.25 mmol) were dissolved in the above solution and stirred at room temperature for 30 min. Subsequently, the mixture was added into a high-pressure hydrothermal reactor and then transferred to a preheated oven at 180 ℃. After 24 h, the mixture was taken out and cooled to room temperature. Finally, the product was centrifuged (15000 rpm), washed three times with deionized water, and then freeze-dried for 12 h.

***In vitro* catalytic activity of FeRu nanozymes**

**The consumption of H_2_O_2_**

The consumption of H_2_O_2_ was detected by using ammonium titanyl oxalate as a probe. In brief, gradient concentrations of FeRu nanozymes (0, 25, 50, 100, 150, 200, 300 μg·mL^-1^) were added to PBS (pH 6.5), followed by the addition of H_2_O_2_ (50 μL, 0.1 M). After reaction for 3 h, ammonium titanyl oxalate (200 μL, 10 mg·mL^-1^, H_2_O) were added. The residual content of H_2_O_2_ was detected by the UV-vis absorption spectrum at 360 nm. Moreover, FeRu nanozymes (150 μL, 1 mg·mL^-1^) and H_2_O_2_ (50 μL, 0.1 M) were added to PBS (2.6 mL, pH 6.5). Subsequently, ammonium titanyl oxalate (200 μL, 10 mg·mL^-1^, H_2_O) was added at different time points (0, 1, 2, 3, 4, 5, 6, 7, 8 h), respectively. The residual H_2_O_2_ was detected by the method mentioned above.

**The consumption of GSH**

The residual amount of GSH was detected using Ellman's reagent (5,5'- dithiobis (2-nitrobenzoic acid), DTNB). Briefly, an aliquot of GSH solution (100 μL, 1 mg·mL^-1^) was added to the FeRu nanozymes solution (0, 40, 80, 120, 160, 200, and 300 μg·mL^-1^) and evenly mixed, respectively. After 2 h, the mixture was filtered through a disposable needle filter to obtain a clear solution. This filtrate was then mixed with DTNB solution (100 μL, 1 mg·mL^-1^) and PBS (1.9 mL, pH 6.5). The absorption value of TNB at 412 nm was detected using UV-vis absorption spectrometry.

**Detection of CAT-like activity *in vitro***

Firstly, the formation of bubbles on the wall of the reaction tube was observed. The experiment was divided into four groups: Ⅰ) control (PBS, pH 6.5), Ⅱ) PBS+H_2_O_2_ (100 mM), Ⅲ) PBS+ FeRu (100 μg·mL^-1^), Ⅳ) PBS+ H_2_O_2_+FeRu (100 mM, 100 μg·mL^-1^). After 10 minutes of reaction, the bubbles on the tube wall were captured and recorded by the camera.

Subsequently, the production of oxygen was observed using the oxygen indicating probe, [Ru(pdd)_3_]Cl_2_, and the CAT-like activity of FeRu nanozymes at different substrate concentrations was assessed. Specifically, 200 μL of reaction solution containing 5 μM [Ru(pdd)_3_]Cl_2_ was prepared by PBS at pH 6.5. FeRu nanozymes (100 μg·mL^-1^) and H_2_O_2_ of various concentrations (0, 5, 10, 50, 100 mM) were added and incubated in 96-well plate for 20 min at 37°C in dark condition. Furthermore, the CAT-like activity of different concentrations of FeRu nanozymes in vitro was then measured. mM H_2_O_2_ and different concentrations of FeRu nanozymes (0, 25, 50, 100, 150, 200 μg·mL^-1^) were mixed with the above reaction solution and incubated in 96-well plate at 37°C for 20 min. Then the fluorescent intensity of [Ru(pdd)_3_]Cl_2_ at 620 nm was detected using a multifunctional microplate reader.

The catalytic production of oxygen by different concentrations of nanozymes was measured by a dissolved oxygen meter. PBS reaction system of 10 mL pH 6.5 with different concentration of nano-enzyme was set up. First, different amounts of FeRu nanozymes were added to PBS at concentrations of 0, 20, 50, 100, 150, 200 μg·mL^-1^, and then H_2_O_2_ (50 mM) was added, and the timing began immediately, recording the dissolved oxygen concentration in the solution every 10 s. To assess the CAT-like catalytic efficiency under biomimetic conditions, the same experiment was conducted using PBS containing 10% FBS.

**Detection of POD-like activity *in vitro***

Methylene blue was used as the probe to detect ·OH produced by the consumption of H_2_O_2_. Briefly, 3 mL of the mixed solution containing FeRu nanozymes (100 μg·mL^-1^), H_2_O_2_ (100 μL, 10 M), and MB (20 μL, 10 μg·mL^-1^, H_2_O) was prepared in PBS at pH 6.5. The UV-vis absorption of methylene blue (MB) at 664 nm was measured at different time points (1, 5, 10, 15, 20, 25, 30, and 60 min).

**CAT-mimic activity and kinetic assay**

The oxygen produced by FeRu nanozymes was quantified using a dissolved oxygen meter. The reaction system was 10 mL PBS buffer at pH 6.5. First, an equal amount of FeRu nanozymes (100 μg·mL^-1^) was added to the buffer, and then gradient concentration of H_2_O_2_ (0, 10, 20, 50, 100, 150, 200 mM) was added to each group, among which 0 mM group was a blank control group and was not involved in subsequent calculation. After the addition of H_2_O_2_, the timing began, the concentration of dissolved oxygen in the solution was recorded every 10 s. For each H_2_O_2_ concentration, the initial reaction rates (*V_0_*) were calculated. The rates were plotted against H_2_O_2_ content and then fitted with the Michaelis-Menten curve (Equation 2). Furthermore, a linear double-reciprocal plot (Lineweaver-Burk plot, Equation 3) was used to determine the *K_M_* and *V_max_*. ([S] represents the substrate concentration)

**POD-mimic activity and kinetic assay**

POD-mimic activity assays of FeRu nanozymes were performed using H_2_O_2_ as the substrate in the presence of TMB. Briefly, FeRu nanozymes (10 μg·mL^-1^), H_2_O_2_ (0, 1, 2, 3, 4, 5, 6, 7, 8, 9, 10 mM), and TMB (20 μL, 5 mg·mL^-1^) were dispersed in 3 mL of PBS solution (pH 6.5). The absorbance was recorded at different time points using a UV-vis spectrophotometer (λ = 652 nm). For each H_2_O_2_ concentration, the initial reaction rates (*V_0_*) were calculated using the Beer-Lambert Law (Equation 1) (*ε*: 39000 M^-1^ cm^-1^ for oxTMB. The rates were plotted against H_2_O_2_ content and then fitted with the Michaelis-Menten curve (Equation 2). Furthermore, a linear double-reciprocal plot (Lineweaver-Burk plot, Equation 3) was used to determine the *K_M_* and *V_max_*. ([S] represents the substrate concentration)

A＝*εlc* (Equation 1)

*V_0_*=$\frac{V_{max}\cdot[S]}{K_{M}+[S]}$ (Equation 2)

$\frac{1}{V_{0}}$=$\frac{K_{M}}{V_{max}}$ · $\frac{1}{[S]}$ + $\frac{1}{V_{max}}$ (Equation 3)

**GSHOx-mimic activity and kinetic assay**

Specifically, FeRu nanozymes（500 μg·mL^-1^）, GSH（0.25、0.5、1、1.5、2 mM）and DTNB（100 μg·mL^-1^） were dispersed into PBS solution (3 mL, pH 6.5). The absorbance was measured at different time points（λ = 412 nm）. For each GSH concentration, the initial reaction rates (*V_0_*) were calculated from the absorbance changes using the Beer-Lambert Law (Equation 1) (*ε*: 13600 M^-1^ cm^-1^ for TNB). The rates were then plotted against GSH content and fitted with the Michaelis-Menten curves (Equation 2). Additionally, a linear double-reciprocal plot (Equation 3) was utilized to calculate the *K_M_* and *V_max_*.

**Computational Method**

All the spin-polarized DFT calculations were conducted based on the Vienna Ab-inito Simulation Package (VASP) [1, 2]. The electron-ion interactions were described by the Projected Augmented-Wave (PAW) potentials, while the exchange-correlation interactions were calculated by employing the Perdew-Burke-Enzerhof (PBE) functional of Generalized Gradient Approximation (GGA) [3, 4]. The vdW-D3 method developed by Grimme was employed to describe the van der Waals interaction [5]. The plane-wave energy cutoff was set as 450 eV. The convergence threshold was set as 1.0×10^-5^ eV in energy and 0.02 eV per Angstrom in force. A vacuum layer of 15 Å was adopted for slab models to avoid the periodic interactions. The Brillouin zone was modeled by gamma centered Monkhorst-Pack scheme, in which a 1×1×1 grid was adopted for all slab models.

The change in the Gibbs free energy of each reaction was calculated (Equation 4).

$\text{ΔG=Δ}\text{E}_{\text{pot}}\text{+Δ}\text{E}_{\text{ZPE}}\text{-TΔS}$ (Equation 4)

In which the ΔE_pot_, ΔE_ZPE_ and the ΔS were referred to as the change in potential energy, the change in the zero-point energy and the change in the entropy.

The zero-point energy was calculated by the summation of all vibrational frequencies (Equation 5).

$E_{\text{ZPE}}=\frac{1}{2}\sum h\upsilon$ (Equation 5)

where the ν corresponded to the vibriational frequency of each normal mode [6].

**Cell culture**

The mouse melanoma cell line (B16) was purchased from the Cell Bank of the Chinese Academy of Sciences (Shanghai, China). B16 cells were maintained in RPMI 1640 medium supplemented with 10% FBS and 1% antibiotics, and cultured in a 5% CO_2_ incubator at 37 ℃。

***In vitro* cytotoxicity analysis**

In vitro cytotoxicity of FeRu nanozymes was measured on B16 cells by MTT assay. Briefly, B16 cells (3×10^4^ cells per well, 100 μL of RPMI 1640 medium) were added into 96-well plates and incubated for 24 h. A series of concentrations of different formulations are added to the above medium for further incubation: Ⅰ) Control (PBS), Ⅱ) CAT (100、200、300、400、500 U·mL^-1^), Ⅲ) BSO (50、100、150、200、250 μg·mL^-1^), Ⅳ) CAT+ FeRu nanozymes, Ⅴ) BSO+ FeRu nanozymes, Ⅵ) FeRu nanozymes (100、200、300、400、500 μg·mL^-1^), respectively. Then, the cells were further cultivated for 24 h evaluated by MTT assay. The same experimental protocol was used to treat breast cancer 4T1 cells to verify the universality of the anti-tumor effect of nanozyme. The same method was used to treat mouse fibroblast L929 cells to verify the selectivity of toxicity.

**Flow cytometry analysis**

B16 cells were planted in 12-well culture plates (1 × 10^5^ cells per well, 1 mL of RPMI 1640 medium) and treated with Control (PBS), CAT (600 U·mL^-1^), BSO (100 μg·mL^-1^), CAT+ FeRu nanozymes, BSO+ FeRu nanozymes, FeRu nanozymes (400 μg·mL^-1^), respectively.

For intracellular ROS level analysis, after 20 h, cells were collected in serum-free medium, washed with PBS and then incubated in DCFH-DA (1 mL, 10 μM) at 37 ℃ for 20 min in the dark.

For mitochondrial membrane potential analysis, after 24 h, cells were collected in serum-free medium, washed with PBS and then incubated in Rhodamine 123 (1 mL, 10 μg·mL^-1^) at 37℃ for 20 min in the dark.

Subsequently, the cells from different groups were washed in serum-free medium, collected and detected by flow cytometry.

**Cell imaging**

The cellular fluorescent images were captured by an inverted fluorescence microscope system or CLSM. B16 cells were planted in 12-well culture plates (1 × 10^5^ cells per well, 1 mL of RPMI 1640 medium) and treated with Control (PBS), CAT (600 U·mL^-1^), BSO (100 μg·mL^-1^), CAT+ FeRu nanozymes, BSO+ FeRu nanozymes, FeRu nanozymes (400 μg·mL^-1^), respectively.

For live-dead cell analysis, after 36 h, the cells were stained with Calcein-AM and PI, respectively.

For intracellular O_2_ detection, after 20 h, B16 cells were stained with [Ru(pdd)_3_]Cl_2_ (7 μM) for 1 h, followed by nuclear staining with Hoechst 33342 (1 μg·mL^-1^) for 30 min.

For intracellular ROS detection, after 20 h, the cells were stained with DCFH-DA (10 μM) for 20 min, followed by nuclear staining with Hoechst 33342 (1 μg·mL^-1^) for 30 min.

For mitochondrial membrane potential measurement, after 24 h, B16 cells were stained with Rhodamine 123 (10 μg·mL^-1^) for 20 min, followed by nuclear staining with Hoechst 33342 (1 μg·mL^-1^) for 30 min.

For cellular LPO detection, after 24 h, B16 cells were stained with BODIPY ^581/591^ C11 (10 μM) for 45 min, followed by fixation with 4% PFA and staining with Hoechst 33342 (1 μg·mL^-1^) for 30 min.

For intracellular DNA damage assessment, after 24 h, then DNA damage assay kit by γ-H2AX immunofluorescence was employed to evaluate the extent of DNA damage in the cells.

For intracellular immune activation, after 16 h, B16 cells were sequentially blocked, incubated with primary antibodies (HMGB1 or CRT antibody), and secondary antibody (Alexa Fluor 555-labeled Donkey Anti-Rabbit IgG (H+L)), followed by staining with DAPI (500 μL).

For disruption analysis of cell membrane, after 24 h, the cell membrane integrity detection kit was utilized to assess the membrane integrity of the treated B16 cells.

**Detection of intracellular markers**

B16 cells were planted in 6-well culture plates and exposed to various treatments including Control (PBS), CAT (600 U·mL^-1^), BSO (100 μg·mL^-1^), CAT+ FeRu nanozymes, BSO+ FeRu nanozymes, FeRu nanozymes (400 μg·mL^-1^), respectively.

For detecting intracellular MDA, after 24 h, the intracellular level of MDA was measured using lipid peroxidation (MDA method) assay kits. Furthermore, the protein concentration in the cell samples was determined using the BCA protein assay kits to calculate the initial MDA content in the samples.

After 16 h, the extracellular ATP levels were measured using ATP assay kits. The chemiluminescence intensity was quantified using a multimode reader.

After 16 h, the total antioxidant capacity of the treated B16 cells was assessed using total antioxidant capacity assay kits. Furthermore, the protein concentration in the cell samples was determined using the BCA protein assay kits to calculate the total antioxidant content in the initial samples.

B16 cells were planted in 6-well culture plates and exposed to Control (PBS), FeRu nanozymes (50, 100, 150, 200, 300 μg·mL^-1^), respectively. After 12 h, the intracellular GSH content in the treated B16 cells was measured using the GSH&GSSG assay kit.

Bio-transmission Electron Microscopy (Bio-TEM): B16 cells were planted in 6-well culture plates and treated with: (a) Control (PBS); (b) FeRu nanozymes (400 μg·mL^-1^) for 12 h. Afterwards, the cells were collected and immobilized for Bio-TEM imaging.

**Western blot assays**

B16 cells were planted in 6-well culture plates and treated with Control (PBS), CAT (600 U·mL^-1^), BSO (100 μg·mL^-1^), CAT+ FeRu nanozymes, BSO+ FeRu nanozymes, FeRu nanozymes (400 μg·mL^-1^), respectively. After 6 h, the cells were collected using trypsin and lysed in a lysis buffer. BCA protein assay was performed to determine the protein content. Subsequently, the protein lysates were separated by SDS-polyacrylamide gel electrophoresis, transferred to a nitrocellulose membrane, and blocked with 5% skim milk. After incubation with the primary antibodies (anti-GPX4 antibody, anti-HIF-1α) and secondary antibodies, protein bands were visualized and analyzed using GraphPad Prism Software.

**Animal**

All animal protocols were granted approval by the Animal Research Committee of Shenyang Pharmaceutical University (No: SYPU-IACUC-S2025-0430-105). The C57BL/6 mice (6-8 weeks old) were purchased from Changsheng Biotechnology Co. Ltd (Benxi, China).

***In vivo* anti-tumor effect**

B16 cells (1×10^6^ cells per mouse) were subcutaneously injected into the right flanks of C57BL/6 mice to establish B16 tumor bearing models. Subsequently, the tumor-bearing mice were divided into three parts for experiments. The dosage of FeRu nanozymes for mice was determined to be 10 mg/kg based on the preliminary results and relevant literature [7, 8, 9].

The first part of mouse, administrated at different frequencies, were randomly divided into three groups (n=5): Ⅰ) Control (PBS), Ⅱ) intratumoral injections of FeRu nanozymes（10 mg·kg^-1^）once every 2 days, Ⅲ) intratumoral injections of FeRu nanozymes（10 mg·kg^-1^）once every 4 days.

The next part of mouse administrated with different formulations were randomly divided into six groups (n=5): Ⅰ) Control (PBS), Ⅱ) CAT（1.5 mg·kg^-1^）, Ⅲ) BSO（1mg·kg^-1^）, Ⅳ) CAT+ FeRu nanozymes, Ⅴ) BSO+ FeRu nanozymes, Ⅵ) FeRu nanozymes（10 mg·kg^-1^）. These formulations were intratumorally injected on days 0, 2, 4, 6, 8, and 10.

The last part of the mouse administrated with FeRu nanozymes combined with αPD-L1 were randomly divided into four groups (n=5): Ⅰ) Control (PBS), Ⅱ) αPD-L1（1 mg·kg^-1^）, Ⅲ) FeRu nanozymes（10 mg·kg^-1^）, Ⅳ) FeRu nanozymes + αPD-L1. Among them, FeRu nanozymes was intratumorally injected on days 0, 2, 4, 6, 8, and 10. αPD-L1 was intratumorally injected on days 1, 3, 5, 7, 9, and 11. The tumor volume and body weight of each mouse was recorded every other day, and tumor volume was calculated using the formula V = width^2^ × length/2. After 12 days of treatment, the tumors were harvested, and the tumor weight was recorded.

***In vivo* distribution of FeRu nanozymes**

To investigate the distribution and metabolism of the nanozymes *in vivo*, near infrared fluorescence imaging was performed. Briefly, tumor-bearing mice were randomly divided into two groups: (1) free DiR, and (2) DiR-labeled FeRu nanozymes group (DiR+FeRu). The formulations were administered via intratumoral injection. Whole-body imaging was conducted at 0 h, 6 h, 12 h, and 24 h post-injection using a small animal *in vivo* imaging system. At the 0 h, 6 h, 12 h, and 24 h time points, the mice were euthanized, and major organs (heart, liver, spleen, lung, kidney) together with the tumor were collected. The harvested tissues were gently rinsed with PBS and placed in a Petri dish for *ex vivo* fluorescence imaging using the same imaging system. Fluorescence quantification of the excised tissues was subsequently performed using a microplate reader (*E_x_/E_m_* = 748/780 nm).

**Blood hematology analysis**

The serum of mice was collected after different treatments. The serum levels of TNF-α, IFN-γ, IL-6 and IL-12p70 were measured using an enzyme-linked immunosorbent assay (ELISA) kits according to the manufacturer's instructions.

**Histology analysis**

At the end of therapeutic period, tumor tissues of mice in each treatment group were collected, fixed with 4% paraformaldehyde, and embedded in paraffin for one day and cut with a microtome. The sections were then stained with hematoxylin and eosin (H&E), TdT-mediated dUTP nick-end labeling (TUNEL), Ki-67 antibody, HIF-1α antibody, CD4 antibody, CD8 antibody, GPX4 antibody, HMGB1 antibody, and CRT antibody. Finally, the slices were observed using an inverted fluorescence microscope.

**Flow cytometric analysis of immune cells**

At the end of the treatment, to analyze the infiltrating lymphocytes, the tumor tissue and spleens were collected and digested in HBSS (Hanks’ Balanced Salt Solution) containing 1.5 mg·mL^-1^ collagenase at 37°C for 1 h to obtain the single-cell suspension. For subsequent flow analysis of lymphocytes, a portion of the single-cell suspension was isolated using lymphocyte separation solution to obtain the lymphocyte suspension. To analyze the frequencies of CD8^+^ T cells (CD3^+^CD4^-^CD8^+^), CD4^+^ T cells (CD3^+^CD4^+^CD8^-^), *T_reg_* cells (CD3^+^CD4^+^Foxp3^+^), DCs (CD11c^+^CD80^+^CD86^+^), *T_cm_* cells (CD44^+^CD62L^+^), NK cells (CD45^+^CD3^+^NK1.1^+^), B cells (CD45^+^CD3^+^CD19^+^), the cells were stained with corresponding antibodies, including anti-CD3-FITC, anti-CD4-Percp-Cy5.5, anti-CD8-APC, anti-Foxp3-PE, anti-CD11c-BV421, anti-CD80-APC, anti-CD86-PE, anti-CD62L-APC, anti-CD44-PE, anti-CD45-Cy5.5, anti-NK1.1-PE, and anti-CD19-APC. The stained cells were analyzed via flow cytometry.

***In vivo* biosafety evaluation**

Healthy C57BL/6 mice were randomly divided into 6 groups (n = 3 / group): Ⅰ) Control, Ⅱ) CAT (1.5mg·kg^-1^), Ⅲ) BSO (1mg·kg^-1^), Ⅳ) CAT+ FeRu nanozymes, Ⅴ) BSO+ FeRu nanozymes, Ⅵ) FeRu nanozymes (10 mg·kg^-1^). The method of administration is subcutaneous injection on the buttocks. The blood samples were collected and centrifuged at 3000 rpm for 10 min to collect serums. For the analysis of serum creatinine (Crea), direct bilirubin (DBIL), total bilirubin (TBIL), aspartate aminotransferase (AST), ureaphil (Urea), creatine kinase (CK), and alanine transaminase (ALT). At the same time, major organs of the mice were dissected for further analysis.

For long-term safety, the mice were subcutaneously injected with FeRu nanozymes (10 mg·kg^-1^) in the right buttock, and the above experiments were conducted at 30 and 45 days after the injection, respectively.

**Statistical analysis**

All data were analyzed using Origin software and GraphPad Prism software. The quantitative data analysis was presented as mean ± standard deviation. A two-tailed Student’s t-test was conducted to statistically analyze the difference between the two groups.

**Supplementary figures**

**Figure S1.** The full-spectrum analysis of elemental composition of FeRu nanozymes.

**Figure S2.** The stability of nanozymes dispersed in PBS (pH 6.5) solutions stored at room temperature for one week (n=3).

**Figure S3.** The zeta potential of FeRu nanozymes (n=3).

**Figure S4.** The XRD spectra of FeRu nanozymes.

**Figure S5.** (a) Concentration-dependent and (b) time-dependent H_2_O_2_ consumption by FeRu nanozymes.

**Figure S6**. The relative fluorescence value of O_2_ generation catalyzed by FeRu nanozymes with (a) different concentrations of H_2_O_2_ (n=4 **P < 0.01, ****P < 0.0001.) and (b) different concentrations of FeRu nanozymes (n=5 **P < 0.01, ****P < 0.0001.).

**Figure S7.** The O_2_ production catalyzed by FeRu nanozymes at different concentrations in PBS containing 10% FBS. (n=3)

**Table S1**

Comparative analysis of CAT-like kinetic parameters among FeRu nanozymes, natural catalase, and other reported nanozymes.

| Nanozymes | *K_m_* (mM) | *V_max_* (mg·L^-1^·s^-1^) | Conditions (pH) | References |
| --- | --- | --- | --- | --- |
| ^a^Fe^3+^/AMP | 112.2 | 0.079 | 6.9 | [10] |
| ^b^ACPP | 70.73 | 0.108 | - | [11] |
| ^c^MCCP | 2.02 | 0.152 | 5.0 | [12] |
| ^d^catalase | 28.8 | - | 7 | [13] |
| FeRu | 135.841 | 0.209 | 6.5 | This work |

Where *K_m_* is the Michaelis-Menten constant that the affinity between enzyme and substrate, *V_max_* is the maximal reaction velocity, condition refers to the pH of the system in which the reaction takes place.

^a^Fe^3+^/AMP: Fe^3+^ and adenosine monophosphate (AMP) formed coordination nanoparticles with comparable performance as natural catalase at ambient conditions for degrading H_2_O_2_.

^b^ACPP: a trimetallic alloy nanozyme AuCuPt-PpIX.

^c^MCCP: an L-cysteine (L-Cys) coordination-driven self-assembly strategy to activate polyvinylpyrrolidone (PVP)-modified Cu single-atom nanozymes MoOx-Cu-Cys.

^d^catalase: catalase derived from bovine liver.

**Table S2**

Comparative analysis of POD-like kinetic parameters among FeRu nanozymes, natural peroxidase, and other reported nanozymes.

| Nanozymes | *K_m_* (mM) | *V_max_* (M·s^-1^) | Conditions (pH) | References |
| --- | --- | --- | --- | --- |
| ^e^Ru1000 | 18.4 | 1.16×10^-6^ | 6.5 | [14] |
| ^f^Fe SAEs | 0.243 | 8.25×10^-10^ | - | [15] |
| ^g^Fe_3_O_4_MNPs | 154 | 9.78×10^-8^ | 3.5 | [16] |
| ^h^Fe_3_O_4_@MoS_2_ | 1.39 | 1.63×10^-7^ | 4.0 | [17] |
| ^i^HRP | 3.7 | 0.871 |  | [18] |
| FeRu | 5.308 | 4.301×10^-7^ | 6.5 | This work |

Where *K_m_* is the Michaelis-Menten constant that the affinity between enzyme and substrate, *V_max_* is the maximal reaction velocity, condition refers to the pH of the system in which the reaction takes place.

^e^Ru1000: the Ru-based nanozyme (Ru1000) with a lattice expansion of 5.99%.

^f^Fe SAEs: a heterogeneous single iron atom catalyst.

^g^Fe_3_O_4_MNPs: magnetite nanoparticles.

^h^Fe_3_O_4_@MoS_2_: Fe3O4@MoS2-Ag nanozyme with defectrich rough surface.

^i^HRP: Horseradish peroxidase.

**Figure S8.** The (a) standard curve and (b) residual concentration diagram of GSH in vitro.

**Table S3**

Comparative analysis of GSHOx-like kinetic parameters among FeRu nanozymes, natural glutathione oxidase, and other reported nanozymes.

| Nanozymes | *K_m_* (mM) | *V_max_* (M·s^-1^) | Conditions (pH) | References |
| --- | --- | --- | --- | --- |
| ^j^FeCo/Fe-Co DAzyme | 1.775 | 1.07×10^-6^ | - | [19] |
| Ru1000 | 0.2 | 8.7×10^-6^ | 6.5 | [14] |
| ^k^Bi_2_Fe_4_O_9_ | 1.85±0.27 | 0.92±0.07×10^-6^ | 7.4 | [20] |
| FeRu | 1.558 | 5.175×10^-8^ | 7.4 | This work |

Where *K_m_* is the Michaelis-Menten constant that the affinity between enzyme and substrate, *V_max_* is the maximal reaction velocity, condition refers to the pH of the system in which the reaction takes place.

^j^FeCo/Fe-Co DAzyme: lipoxygenase (LOX) and phospholipase A2 (PLA2)-co-loaded FeCo/Fe−Co dual-metal atom nanozyme.

^k^Bi_2_Fe_4_O_9_: Bi_2_Fe_4_O_9_ nanosheets.

**Figure S9.** (a), (b) Cytotoxicity profiles of 4T1 cells after different treatments. (n=3, ****P < 0.0001, ns, no significant.)

**Figure S10.** (a), (b) Cytotoxicity profiles of L929 cells after different treatments. (n=3, *P < 0.05, ns, no significant.)

**Figure S11.** MFI of DCFH-DA in B16 cells after various treatments: (n=3, ****P < 0.0001.)

**Figure S12.** MFI of BODIPY^TM^ 581/591 C^11^ in B16 cells with various treatments: (n=3, ****P < 0.0001.)

**Figure S13.** MFI of T11 in B16 cells after various treatments: (n=3, ****P < 0.0001.)

**Figure S14.** MFI of HMGB1 in B16 cells with various treatments: (n=3, ****P < 0.0001.)

**Figure S15.** MFI of CRT in B16 cells with various treatments: (n=3, ****P < 0.0001.)

**Figure S16.** The growth curves of tumor after different treatment frequency.

**Figure S17.** Quantitative distribution of DiR and DiR+FeRu in tumors and major organs at various time points after intratumoral injection. (n=3, *P < 0.05, **P < 0.01, ***P < 0.001, ****P < 0.0001, ns, no significant.)

**Figure S18.** The growth curves of tumor after different treatment combination

**Figure S19.** Representative FCM profiles of (a) DCs, (b) CD8^+^Tcells, (c) CD4^+^Tcells and (d) *T_regs_* in the tumors with various treatment.

**Figure S20**. The immunofluorescence images of TUNEL, H&E staining and Ki67 staining in tumor tissues.

**Figure S21.** The growth curves of tumor after combination treatment with α PD-L1.

**Figure S22.** The heat map of intratumoral levels of TNF-α, IFN-γ, IL-6 and IL-12 after different treatments.

**Figure S23.** The gating strategy of mature CD4 in tumors after various treatments, wherein matured CD4 were denoted as CD3^+^CD4^+^ cells.

**Figure S24.** The gating strategy of mature CD8 in tumors after various treatments, wherein matured CD8 were denoted as CD3^+^CD8^+^ cells.

**Figure S25.** The gating strategy of mature DCs in tumors after various treatments, wherein matured DCs were denoted as CD11c^+^CD80^+^CD86^+^ cells.

**Figure S26.** The gating strategy of mature *T_cm_* in tumors after various treatments, wherein matured *T_cm_* were denoted as CD3^+^CD44^+^ CD62L^+^cells.

**Figure S27.** The gating strategy of mature Treg in tumors after various treatments, wherein matured Treg were denoted as CD3^+^CD4^+^ Foxp3^+^cells.

**Figure S28.** The gating strategy of mature NK cells in tumors after various treatments, wherein matured NK cells were denoted as CD45^+^CD3^+^ NK1.1^+^cells.

**Figure S29.** The gating strategy of mature B cells *i*n tumors after various treatments, wherein matured B cells were denoted as CD45^+^CD3^+^ CD19^+^cells.

**Figure S30.** The assessment of main organs of mice with various treatments after 12 days by H&E staining.

**Figure S31.** Biochemical analysis of the serum of mice with various treatments after 12 days: (a) AST; (b) AST; (c) TBIL; (d) DBIL; (e) Urea; (f) Crea; (g) CK. (n=3, ns, no significant.)

**References**

[1] J. Nai, J. Zhang, X. W. (David) Lou, Chem 2018, 4, 1967.

[2] S. Wang, W. Huo, H. Feng, X. Zhou, F. Fang, Z. Xie, J. K. Shang, J. Jiang, Small 2022, 18, 2203713.

[3] J. Mahmood, F. Li, S.-M. Jung, M. S. Okyay, I. Ahmad, S.-J. Kim, N. Park, H. Y. Jeong, J.-B. Baek, Nature Nanotech 2017, 12, 441.

[4] B. W. MacNabb, X. Chen, S. Tumuluru, J. Godfrey, D. N. Kasal, J. Yu, M. L. M. Jongsma, R. M. Spaapen, D. E. Kline, J. Kline, Immunity 2022, 55, 982.

[5] G. Kresse, J. Furthmüller, Computational Materials Science 1996, 6, 15.

[6] G. Kresse, J. Furthmuller, .

[7] Y. Zou, B. Jin, H. Li, X. Wu, Y. Liu, H. Zhao, D. Zhong, L. Wang, W. Chen, M. Wen, Y.-N. Liu, ACS Nano 2022, 16, 21491.

[8] J. Zhou, D. Xu, G. Tian, Q. He, X. Zhang, J. Liao, L. Mei, L. Chen, L. Gao, L. Zhao, G. Yang, W. Yin, G. Nie, Y. Zhao, J. Am. Chem. Soc. 2023, 145, 4279.

[9] B. Guo, F. Yang, L. Zhang, Q. Zhao, W. Wang, L. Yin, D. Chen, M. Wang, S. Han, H. Xiao, N. Xing, Advanced Materials 2023, 35, 2212267.

[10] M. Jiao, Z. Li, X. Li, Z. Zhang, Q. Yuan, F. Vriesekoop, H. Liang, J. Liu, Chemical Engineering Journal 2020, 388, 124249.

[11] J. Liu, S. Dong, S. Gai, Y. Dong, B. Liu, Z. Zhao, Y. Xie, L. Feng, P. Yang, J. Lin, ACS Nano 2023, 17, 20402.

[12] J. Zhou, D. Xu, G. Tian, Q. He, X. Zhang, J. Liao, L. Mei, L. Chen, L. Gao, L. Zhao, G. Yang, W. Yin, G. Nie, Y. Zhao, J. Am. Chem. Soc. 2023, 145, 4279.

[13] M. Chang, Z. Hou, M. Wang, C. Yang, R. Wang, F. Li, D. Liu, T. Peng, C. Li, J. Lin, Angew Chem Int Ed 2021, 60, 12971.

[14] S. Zhong, Nature Communications 2024.

[15] C. Zhao, C. Xiong, X. Liu, M. Qiao, Z. Li, T. Yuan, J. Wang, Y. Qu, X. Wang, F. Zhou, Q. Xu, S. Wang, M. Chen, W. Wang, Y. Li, T. Yao, Y. Wu, Y. Li, Chem. Commun. 2019, 55, 2285.

[16] L. Gao, J. Zhuang, L. Nie, J. Zhang, Y. Zhang, N. Gu, T. Wang, J. Feng, D. Yang, S. Perrett, X. Yan, nature nanotechnology 2007, 2.

[17] F. Wei, X. Cui, Z. Wang, C. Dong, J. Li, X. Han, Chemical Engineering Journal 2021, 408, 127240.

[18] S. Osman, C. Peng, F. Li, H. Chen, J. Shen, Z. Zhong, W. Huang, D. Xue, J. Liu, Advanced Science 2022, 9, 2205575.

[19] Y. Liu, R. Niu, R. Deng, S. Song, Y. Wang, H. Zhang, J. Am. Chem. Soc. 2023, 145, 8965.

[20] Y. Zou, B. Jin, H. Li, X. Wu, Y. Liu, H. Zhao, D. Zhong, L. Wang, W. Chen, M. Wen, Y.-N. Liu, ACS Nano 2022, 16, 21491.
